# Immune and inflammatory insights in atherosclerosis: development of a risk prediction model through single-cell and bulk transcriptomic analyses

**DOI:** 10.3389/fimmu.2024.1448662

**Published:** 2024-09-19

**Authors:** Xiaosan Chen, Zhidong Zhang, Gang Qiao, Zhigang Sun, Wei Lu

**Affiliations:** Heart Center of Henan Provincial People’s Hospital, Central China Fuwai Hospital, Central China Fuwai Hospital of Zhengzhou University, Zhengzhou, Henan, China

**Keywords:** atherosclerotic plaques, single-cell sequencing, macrophages, immuno-inflammatory responses, riskscore model

## Abstract

**Background:**

Investigation into the immune heterogeneity linked with atherosclerosis remains understudied. This knowledge gap hinders the creation of a robust theoretical framework essential for devising personalized immunotherapies aimed at combating this disease.

**Methods:**

Single-cell RNA sequencing (scRNA-seq) analysis was employed to delineate the immune cell-type landscape within atherosclerotic plaques, followed by assessments of cell-cell interactions and phenotype characteristics using scRNA-seq datasets. Subsequently, pseudotime trajectory analysis was utilized to elucidate the heterogeneity in cell fate and differentiation among macrophages. Through integrated approaches, including single-cell sequencing, Weighted Gene Co-expression Network Analysis (WGCNA), and machine learning techniques, we identified hallmark genes. A risk score model and a corresponding nomogram were developed and validated using these genes, confirmed through Receiver Operating Characteristic (ROC) curve analysis. Additionally, enrichment and immune characteristic analyses were conducted based on the risk score model. The model’s applicability was further corroborated by *in vitro* and *in vivo* validation of specific genes implicated in atherosclerosis.

**Result:**

This comprehensive scRNA-seq analysis has shed new light on the intricate immune landscape and the role of macrophages in atherosclerotic plaques. The presence of diverse immune cell populations, with a particularly enriched macrophage population, was highlighted by the results. Macrophage heterogeneity was intricately characterized, revealing four distinct subtypes with varying functional attributes that underscore their complex roles in atherosclerotic pathology. Intercellular communication analysis revealed robust macrophage interactions with multiple cell types and detailed pathways differing between proximal adjacent and atherosclerotic core groups. Furthermore, pseudotime trajectories charted the developmental course of macrophage subpopulations, offering insights into their differentiation fates within the plaque microenvironment. The use of machine learning identified potential diagnostic markers, culminating in the identification of RNASE1 and CD14. The risk score model based on these biomarkers exhibited high accuracy in diagnosing atherosclerosis. Immune characteristic analysis validated the risk score model’s efficacy in defining patient profiles, distinguishing high-risk individuals with pronounced immune cell activities. Finally, experimental validation affirmed RNASE1’s involvement in atherosclerotic progression, suggesting its potential as a therapeutic target.

**Conclusion:**

Our findings have advanced our understanding of atherosclerosis immunopathology and paved the way for novel diagnostic and therapeutic strategies.

## Introduction

Atherosclerosis is a complex disease characterized by the accumulation of lipids and fibrous elements within large arteries and poses a considerable public health challenge globally (1). This disease constitutes a principal causative agent for numerous cardiovascular disorders. These include myocardial infarctions (MI) resulting from coronary artery disease (CAD), strokes originating from cerebrovascular disease (CVD), and limb amputations necessitated by peripheral artery disease (PAD) (2–5). Significant stenosis that restricts blood flow and causes critical tissue hypoxia is the eventual outcome of the chronic accumulation of plaques, which obstruct vessels within the subendothelial intimal layer of large and medium-sized arteries (6). MI and stroke constitute the leading causes of mortality, surpassing even cancer. They are responsible for approximately 31% of deaths in the United States and a similar proportion globally (7). Current clinical guidelines prioritize addressing such complications. Therapies in clinical practice, that aptly inhibit or curtail the progression of atherosclerosis, prominently include the use of drugs that reduce low-density lipoprotein (LDL) cholesterol (8). As the illness swiftly escalates as a leading cause of global morbidity and mortality (9), a deep-rooted understanding of its epidemiology, pathogenesis, and treatment methodologies, along with the advancements in clinical and basic research, is essential.

Atherosclerosis is characterized as a disease typified by the accumulation of excessive cholesterol, initiated through the entrapment of lipoproteins such as low-density lipoprotein (LDL) within the intimal layer of arteries (10). Atherosclerosis is characterized as a disease typified by the accumulation of excessive cholesterol, initiated through the entrapment of lipoproteins such as low-density lipoprotein (LDL) within the intimal layer of arteries (11). Plasma cholesterol, LDL cholesterol, and certain apolipoproteins, particularly Apolipoprotein B (ApoB), show strong associations with clinically apparent atherosclerosis (12–14). Recent studies have demonstrated a significant association between inflammatory responses, immune system activity, and the occurrence and progression of atherosclerosis (15, 16). Atherosclerosis, a chronic inflammatory disorder, is accompanied by a persistent, low-grade inflammatory response that attracts cells from both the innate and adaptive immune systems to the plaque formed in the arteries. Atherosclerotic plaque, in turn, becomes a target for certain cells that specifically recognize ApoB, the central protein of LDL particles. Thus, autoimmune factors also contribute to the manifestation of atherosclerosis as a chronic inflammatory disorder (17, 18). Despite considerable research endeavors, the precise immune mechanisms involved in these phenomena are still largely undetermined. The inherent immune diversity observed within atherosclerotic plaques suggests a noteworthy but obscure role for other immune cells at the locus of plaque development (19). Many studies propose that the circulating immune cells significantly impact the clinical progression of atherosclerosis, where a chronic, low-level inflammatory response accompanies the development of atherosclerosis, attracting cells from the innate and adaptive immune systems to the atherosclerotic plaque (17, 20, 21). Remarkably, patients enduring acute cardiovascular events display greater quantities of circulating monocytes, CD4+ T cell subtypes, and macrophages (22, 23). In addition, aberrant metabolic processes contribute to macrophage mitochondrial dysfunction within the context of atherosclerosis (18, 24). However, substantial knowledge gaps remain concerning the interactions between systemic immune responses and those emerging at the rupture site of the plaque. Therefore, investigating immune cell phenotypes and their functional relationships within both plaques and blood samples drawn from individual patients is vital, yet generally under-researched. These comprehensive notions could potentially lay the groundwork for innovative immunotherapies.

In this study, scRNA-seq was employed to analyze high-quality immune cells from atherosclerotic plaques. Immune cell enrichment in the atherosclerotic core compared to proximal adjacent samples was discovered. Notable immune cell interactions revealed using the MuSiC algorithm and intercellular communication analysis highlighted the importance of macrophages in atherosclerosis pathogenesis. Macrophage heterogeneity was explored, revealing four distinct subtypes with different functional profiles. Macrophage development trajectories were tracked, and vital genes along with subpopulations’ lineage differentiation were identified. Using WGCNA, gene co-expression networks were created to isolate critical genes for further analysis. Gene enrichment studies clarified the biological roles of these genes. Potential atherosclerosis biomarkers were identified using machine learning algorithms, allowing the construction of a diagnostic model with high predictive accuracy. Patients’ risk was further stratified using a developed riskScore model, and molecular as well as immune characteristics associated with different risk levels were investigated. Validation *in vivo* and *in vitro* within atherosclerosis models demonstrated the potential influence of targeted genes on disease pathology.

## Materials and methods

### Acquisition of raw data

Human atherosclerotic plaque single-cell data were sourced from the GEO database, under the accession numbers GSE155512, which encompassed data from three distinct plaques from separate individuals, and GSE159677, detailing both atherosclerotic core (AC) plaques and corresponding proximal adjacent (PA) plaques from three patients. Additional bulk mRNA transcriptomic profiles were retrieved from several repositories: GSE120521, GSE41571, GSE163154, GSE28829, GSE43292, GSE100927, obtained through the GEO database, and E-MTAB-2055, accessed via ArrayExpress. Detailed clinical features and sample specifics across these bulk mRNA datasets were compiled in **Supplementary Table S1**.

In this research, data from three datasets, specifically GSE28829, GSE163154, and GSE43292, were integrated using the “sva” package’s Combat function in R, resulting in a collective of 60 stable and 73 unstable atherosclerotic plaques for the test set, with the exclusion of three outliers. Four other datasets, namely GSE120521, GSE41571, GSE100927, and E-MTAB-2055, were earmarked for validation purposes.

The raw datasets retrieved from the GEO were subjected to preprocessing and normalization employing the Robust Multiarray Average (RMA) method incorporated within the “affy” R package.

### scRNA-seq data processing and cell annotation

Datasets from the GEO repository featuring scRNA-seq data of human arterial plaque were obtained and reprocessed utilizing the “Seurat” R package (version 4.10). We considered genes for analysis if they were present within a minimum of three individual cells. We selected cells that presented a gene count in the range of 200 to 4000, a nCount_RNA that was under 25000, and mitochondrial gene content below 15% to ensure the retention of premium scRNA-seq information. The parameter settings for this single-cell analysis were referenced based on several high-quality researches (25, 26). From this curated dataset, a total of 55,981 cells were deemed appropriate for further examination. Through the application of NormalizeData and ScaleData methodologies, these cells underwent normalization and scaling processes. After this data refinement, utilizing the Seurat toolkit’s FindVariableFeatures function permitted the identification of the superior 3000 variably expressed genes. To mitigate batch variation, potentially compromising subsequent analytical steps, the RunHarmony tool was employed. The discovery of pivotal anchoring points was conducted via principal component analysis. The top 20 principal components were then subjected to examination using the t-SNE approach to unveil significant cellular conglomerates. The subsequent procedure involved the delineation of 17 discrete cell clusters by leveraging the FindNeighbors and FindClusters routines, setting a resolution parameter at 0.35, and then graphically representing these clusters in a t-SNE plot. Each cluster was painstakingly classified into recognized principal cell types based on the expression of canonical marker genes. Finally, to catalog the signature markers for all identified cell groups, we called upon the COSG R package, operating with specified parameters (mu=10, n_genes_user=50).

### Annotating cell types in bulk RNA−seq dataset

The Single Cell Multi-Subject (MuSiC) approach effectively determines cell quantity through deconvolution. By analyzing gene expression exclusive to cell types from single-cell RNA sequencing data, this algorithm discerns the various cellular subpopulations’ relative abundances within the comprehensive dataset GSE100927’s RNA sequencing. A conventional methodology was utilized to deduce the proportions of cell types within mass peripheral blood specimens. Variances across cell populations among the different cohorts were graphically depicted.

### Cell communication analysis

The CellChat objects were created by the “CellChat” R package (https://www.github.com/sqjin/CellChat) (27) based on the UMI count matrix for each group (PA and AC). The database for human ligand-receptor interactions, known as “CellChatDB.human,” was the selected source of data. Cell-to-cell communication analysis was performed utilizing the preset default parameter configuration. To facilitate a comparative analysis of interaction counts and the intensity of these interactions, CellChat data from the respective categories were merged through the “mergeCellChat” utility. The task of portraying variances in either the quantity or intensity of interactions across diverse cellular populations between the examined clusters was accomplished with the help of “compareInteractions” and “netVisual_circle” tools. Subsequently, the expression patterns of signaling genes across different groups were depicted using the “netVisual_bubble” function.

### Trajectory analysis

The Monocle2 algorithm was used to explore the differentiation trajectories of the selected clusters (28). To analyze specific cell clusters, we utilized the ‘subset’ tool within Seurat. Following this, the creation of a CellDataSet object was completed through monocle2’s ‘newCellDataSet’ utility, applying the ‘lowerDetectionLimit’ attribute at a threshold of 0.5. After the initial phase, which included the computation of size factors and dispersion estimates, cells and genes of inferior quality were eliminated. The screening was executed by deploying ‘detectGenes’ and ‘subset’ with the ‘min_expr’ criterion positioned at 0.1. In pursuit of identifying genes with varying levels of expression throughout the cellular trajectory, the ‘differentialGeneTest’ was employed. To condense data into a more manageable form, the ‘reduceDimension’ was used in conjunction with the ‘DDRTree’ technique. Visualization of the ordered cells, the expression of genes over pseudotime, and the gene expression across different branches were achieved through ‘plot cell trajectory’, ‘plot genes in pseudotime’, and ‘plot genes branched heatmap’, respectively. Which, this process was complemented by implementing a CytoTRACE analysis—an unsupervised method that predicts the differentiation continuum within single-cell transcriptomes (29). For visual representation, ‘plotCytoGenes’ and ‘plotCytoTRACE’ were utilized in alignment with the predetermined guidelines of the recommended analytical sequences.

### Estimate the scores of different phenotypes based on the scRNA-seq data

Genetic markers characteristic of various phenotypes — including cholesterol removal, programmed cell death related to iron (ferroptosis), new blood vessel formation (angiogenesis), cell debris ingestion (phagocytosis), cellular component degradation (autophagy), digestive cellular organelle (lysosome) activity, oxygen deprivation (hypoxia), immediate immune response (acute inflammatory response), and stress on the protein-folding cellular department (endoplasmic reticulum stress) — were extracted from the comprehensive Molecular Signatures Database (MSigDB). Following this, to calculate the phenotype-associated scores for different cohorts, we utilized the AUCell tool, adhering to its standard parameters, within the framework provided by the irGSEA software package.

### WGCNA analysis

The “WGCNA” package in R, which implements the WGCNA technique, was employed to construct the gene co-expression network of the GSE100927 dataset. To enhance the precision of the analysis, the top quartile of genes exhibiting high variance was selected as the input data. The procedure was meticulously carried out in several steps. Initially, the “goodSamplesGenes” function was employed to remove any genes with missing values. Subsequently, an optimal soft threshold for calculating the adjacency was determined through visual inspection. The expression matrix was then converted into an adjacency matrix, which was further transformed into a topological overlap matrix (TOM) to delineate the genetic interconnections within the network. To capture the nuances of interconnectedness, average linkage hierarchical clustering was conducted based on the variations observed in the TOM. Dynamic pruning of the hierarchical clustering tree was applied to amalgamate modules with high correlation coefficients, thereby identifying akin modules. The module eigengenes (MEs), representing the collective gene expression of each module, formed the cornerstone of the gene modules. The association between the eigengene values and clinical traits was assessed using Pearson correlation. The final step involved selecting the genes within the module exhibiting the most substantial correlation with macrophages for subsequent investigation.

### Machine learning approaches for feature selection and visualization

Several machine learning techniques, specifically LASSO, SVM-RFE, and the Random Forest (RF) algorithms, were utilized to analyze characteristic methylation-related genes leveraging the R libraries “glmnet,” “e1071,” “caret,” and “Boruta.” The LASSO algorithm identified critical variables by tuning the optimal lambda (λ) value, which was determined through five-fold cross-validation. Meanwhile, the Boruta algorithm pinpointed pertinent genes, obliging to specific criteria (namely 300 iterations and a significance threshold of p < 0.01).

After the removal of non-significant genes, the notable genes discerned through Boruta were integrated into both SVM-RFE and RF analyses, facilitated by the “e1071” and “caret” tools, correspondingly. These analytical models incorporated default settings and hinged on the strength of five-fold cross-validation to curtail the risk of overfitting.

The study cohort, featuring atherosclerotic plaque specimens from diverse datasets, was arbitrarily apportioned into two groups: a training segment (60%) for algorithm tuning, and a validation cohort (40%) for model authentication. Integration of genes identified by each distinct algorithm resulted in the establishment of a definitive set of characteristic genes. To ascertain the diagnostic accuracy of these findings, an evaluation via the receiver operating characteristic (ROC) curve’s area under the curve (AUC) metric was conducted, utilizing the “pROC” R.

### Construction and assessment of a risk model based on characteristic genes

A prognostic nomogram was constructed using macrophage-related distinctive genes, with the ‘rms’ package employed as the foundational tool. To ascertain the precision of the nomogram, a calibration plot was devised. The clinical utility of this nomogram was further assessed through decision curve analysis, conducted via the ‘ggDCA’ package within R.

For patients with atherosclerosis, individual risk scores were calculated drawing on the LASSO machine learning algorithm’s coefficients, aligning to the formula: riskScore = sum (Coefficients_i × Expressioni), wherein ‘i’ indexes the macrophage-related genes incorporated within our risk evaluation model. Patient stratification into high or low-risk cohorts hinged on the median value of these computed risk scores. The predictive performance of the model was examined leveraging ROC curves. The validation of the signature’s predictive capacity was performed using three independent datasets, with the AUC serving as the measurement metric.

### Enrichment analysis

Kyoto Encyclopedia of Genes and Genomes (KEGG) and Gene ontology (GO) enrichment analysis were performed using the previously described “clusterProfiler” R package (30). Biological functions ascribed within gene ontology include three main categories: biological processes (BP), molecular activities (MF), and cellular constituents (CC). P-values under the threshold of 0.05 were deemed to reflect statistical significance.

A Gene Set Variation Analysis (GSVA) was undertaken to scrutinize the diversity present in biological processes and the actions of different pathways. The GSVA package in R was utilized for computations (31). For the analysis of gene set variation, we prioritized hallmark gene collections sourced from the MSigDB repository. To establish distinctions in biological processes and signaling cascade functionality, the “limma” package within the R programming environment was leveraged. Only those outcomes where absolute t-values exceeded a GSVA score of 2 were regarded as having statistical relevance. In addition, an evaluation of gene set enrichment (GSEA) was conducted utilizing the “clusterProfiler” package in R, which facilitated the examination of altered pathway activities (32). To ascertain statistical significance, p-values less than 0.05 were considered. The ranking of Normalized Enrichment Scores (NES) adhered to this criterion. Additionally, the progeny R package was employed to compute activity scores for traditional signaling pathways associated with disease, with comparisons made between different groups. In this context, only p-values falling below the 0.05 threshold were recognized as statistically significant.

### Atherosclerotic immunity

The immune infiltrating levels were conducted using ssGSEA algorithms based on the GSVA package (31). Briefly, the study measured diverse immune cell proportions in each specimen through established marker gene panels. Subsequent computation of immune cell subset compositions or their relative quantities utilized specific analytic algorithms. Group disparities in immune cell penetration were statistically analyzed using the non-parametric Wilcoxon rank-sum test. Heatmaps graphically represented the varying degrees of immune cell presence across atherosclerotic samples under each algorithm’s results. Moreover, the “ESTIMATE” R package was deployed to deduce the immune infiltration in sepsis patients. Furthermore, immune checkpoints consist of a range of molecules (including those for antigen presentation, cell adhesion, co-inhibitory and co-stimulatory interactions, along with ligands and receptors) present on immune cells, crucial for moderating immune reaction magnitude. The research culminated in contrasting the expression of recognized immune checkpoint genes between the compared cohorts.

### Cell culture

The RAW264.7 mouse macrophage cell line was obtained from the Shanghai Cell Bank Type Culture Collection Committee, Shanghai, China. Cells were maintained in Dulbecco’s Modified Eagle Medium (DMEM) (SH30022.01, HyClone, USA) supplemented with 10% fetal bovine serum (10099141C, Gibco, USA), 100 units/mL penicillin, and 100 µg/mL streptomycin (SV30010, HyClone, USA), and incubated at 37°C in a humidified atmosphere with 5% CO2. Oxidized low-density lipoprotein (ox-LDL) was selected as the inducer due to its capability to stimulate endothelial cells and macrophages in the intima of atherosclerotic lesions, prompting them to secrete pro-inflammatory cytokines, chemokines, and adhesion molecules. Upon recognizing and internalizing ox-LDL, monocyte-derived macrophages transform into foam cells, which are key indicators of unstable atherosclerotic plaques. Consequently, ox-LDL-treated RAW264.7 cells were employed as an *in vitro* model to investigate these processes.

### Lentivirus transfection

Before exposure to oxidized low-density lipoprotein (ox-LDL), RAW 264.7 cells were transfected with either rat RNASE1 short hairpin RNA (shRNA) lentivirus or a non-targeting control shRNA lentivirus (Genechem, Shanghai, China) at a multiplicity of infection (MOI) of 5, following the manufacturer’s protocol. Forty-eight hours post-transfection, the cells were utilized for various experiments. The efficiency of RNASE1 knockdown was verified using reverse transcription-quantitative polymerase chain reaction (RT-qPCR). The experimental groups were designated as follows: Control (Con), model (AS), model + non-target control shRNA lentivirus (AS+ lv-shNC), and AS + RNASE1 shRNA lentivirus (LDL+Lv-shRNA). All subsequent *in vitro* experiments were performed a minimum of three times for consistency.

### Evaluation of cell viability

For the MTT assays, RAW 264.7 cells were plated in 96-well plates. Once adhesion was confirmed, the cells were incubated with either 200μL of complete medium (DMEM supplemented with 10% fetal bovine serum) as the normal control (NC) or with complete medium containing 50μg/mL oxidized LDL (ox-LDL) to establish the model (M). Following 24 hours of incubation, the impact of the drugs on cell viability was determined using the MTT assay.

### Migration assay in the cultured RAW 264.7 cells

The motility of the RAW 264.7 cell line—mouse-derived macrophages—was assessed employing transwell inserts of 6.5 mm diameter, which possess 8 µm sized pores (sourced from Corning Costar, NY, USA). These chambers were subjected to preliminary processes, including coating with a Poly L-lysine hydrobromide solution at a concentration of 0.1 mg/mL (Sigma-Aldrich, St. Louis, USA) or a control medium. Following this coating, 1 × 10^5 macrophages in 50 µL of DMEM/F12 with a 1% FBS supplement were introduced to the upper compartment of the transwell by the researchers. An 18-hour interval was allowed for the cells to migrate, after which fixation of the cells was performed using a 4% paraformaldehyde solution for 20 minutes. Any non-migrating cells were gently swabbed from the top face of the membrane using a cotton bud, while the migrated cells, which had adhered to the underside of the transwell membrane, were stained using 0.1% crystal violet for half an hour. For the quantitative analysis, five representative photos from different sections of each membrane—including the center and the four quadrants—were captured via an inverted microscope manufactured by Leica, based in Wetzlar, Germany.

### Animal studies

Male Wistar rats (Beijing Vital River Laboratory Animal Technology), weighing between 170 and 210 grams and aged 8 weeks, were housed in a climate-controlled environment maintained at 22°C with 55% relative humidity. This research adhered to the established guidelines in the Guide for the Care and Use of Laboratory Animals.

### Generation of adenovirus-mediated RNASE1 knockdown rat model via intravenous injection

To further investigate the role of RNASE1 in atherosclerosis, an *in vivo* RNASE1 knockdown model was established. Briefly, adenoviral vectors containing RNASE1-specific short hairpin RNA (shRNA) (Ad-shRNASE1) and a scrambled negative control shRNA (Ad-shNC) were obtained from RiboBio (Guangzhou, China). Each rat received an intravenous injection of approximately 3x10^10 plaque-forming units (PFU) of Ad-shRNASE1 in 200 μL of normal saline, comprising the treatment group. Similarly, the control group was given an equal quantity of Ad-shNC. The knockdown efficiency was quantified by quantitative real-time PCR (qRT-PCR) 14 days post-injection. Total RNA was isolated from the blood samples using a commercially available RNA extraction kit, in accordance with the manufacturer’s protocol. The qRT-PCR analyses were then conducted using specific primers targeting the rat RNASE1 gene to determine its expression levels. Additionally, on day 14, the models were evaluated, and peripheral blood was collected as described.

### Animal treatments

The model of rat atherosclerosis was constructed according to the method of previous studies (33, 34). In brief, following a 7-day acclimatization period, the animals were assigned to four groups: normal control (Con), model (AS), AS+Ad-shRNASE1, and AS+Ad-shNC. The development of the atherosclerosis (AS) model and adenovirus administration were carried out concurrently over 14 weeks. Before the initiation of the model, Vitamin D3 (VD3) at a dose of 600,000 IU/kg was administered intraperitoneally. Throughout the 14 weeks, the animals were maintained on a high-fat diet. At the study’s conclusion, after 14 weeks, the rats were euthanized.

### RTq-PCR

Total RNA was extracted from rat peripheral total blood samples or RAW 264.7 cells using the Trizol reagent (Invitrogen, USA). The extracted RNA was then reverse-transcribed into complementary DNA using the RevertAid First Strand cDNA Synthesis Kit following the manufacturer’s instructions. The quantitative RT-PCR was performed using the Mx3000P QPCR System (Stratagene, La Jolla, CA). The primer sets utilized were as listed: RANSE1:forward 5’-TGCAGGGACTAGGGTAGTGG-3’ and reverse 5’-CATGACACAGGACAGGAACG-3’. CD14: forward: 5’-CACAGCCTAGACCTCAGCCACAAC-3’; reverse: 5’-CCAGCCCAGCGAACG ACAG-3’) To quantify the relative mRNA expression, the cycle threshold (CT) of the target gene was compared to that of β-actin, and the findings were presented as fold changes using the 2-ΔΔCt method.

### ELISA and serum lipid detection

The concentrations of serum total cholesterol (TC), low-density lipoprotein cholesterol (LDL-C), and high-density lipoprotein cholesterol (HDL-C) in cells were quantified using specific ELISA assay kits. Standards and samples were dispensed, followed by the addition of the appropriate reaction reagent. The absorbance was subsequently measured at the designated wavelength, and the concentrations of the samples were calculated using the formula provided in the kit’s instructions.

### Histopathological observations

After the excision of the aorta, the tissues were immediately fixed in a 10% solution of formaldehyde, followed by a gradual dehydration process using ethanol. They were then embedded in paraffin and sectioned into slices with a thickness ranging from 3 to 5 micrometers. After hematoxylin and eosin (H&E) staining, the pathological condition of the rat aortic tissue was evaluated using a light microscope.

### Oil red O staining in lipid-rich plaque of rat aortic sinus

To analyze the lipid-rich plaque in the rat aortic sinus, the freshly obtained cardiac tissues were fixed using a 4% paraformaldehyde solution. The fixation process lasted for a minimum of 48 hours. After that, a gradient dehydration technique was employed using sucrose solutions of concentrations 10%, 20%, and 30%. Optimal cutting temperature (OCT) compound was used to embed the tissues, and subsequently, the hearts were sliced into 10-µm-thick sections using a cryostat. This slicing technique effectively exposed the atrial and aortic sinus regions. Staining the sections was done by immersing them in a solution containing 0.5% Oil Red O in isopropanol, with dilution achieved using di-deionized water (ddH2O) at a ratio of 3:2. The staining process occurred at a temperature of 37°C for 2 hours. To facilitate visualization, hematoxylin was utilized as a counterstain for 2 minutes. For quantification purposes, ImageJ software was employed to measure the areas indicating lipid-rich plaques stained with Oil Red O. Statistical analysis involved calculating the proportion of positively stained regions relative to the overall intimal area.

### Data processing and statistical evaluation

All data and statistical assessments were conducted employing the R computational environment. To examine the association between two continuous metrics, Spearman’s rank correlation method was applied. To assess the disparities in continuous metrics across two distinct groups, we utilized either the Wilcoxon rank-sum test or the two-sided Student’s t-test, contingent upon the data distribution. For analyses involving categorical variables, the chi-square test was the chosen method. Our statistical examinations were uniformly processed through the R platform. Significance thresholds were designated as follows: non-significant (ns) for p-values exceeding 0.05, and levels of significance indicated by *p < 0.05, **p < 0.01, ***p < 0.001, and ****p < 0.0001 for lower p-values.

## Result

### scRNA-seq analysis of atherosclerotic plaques

**Figure 1:**
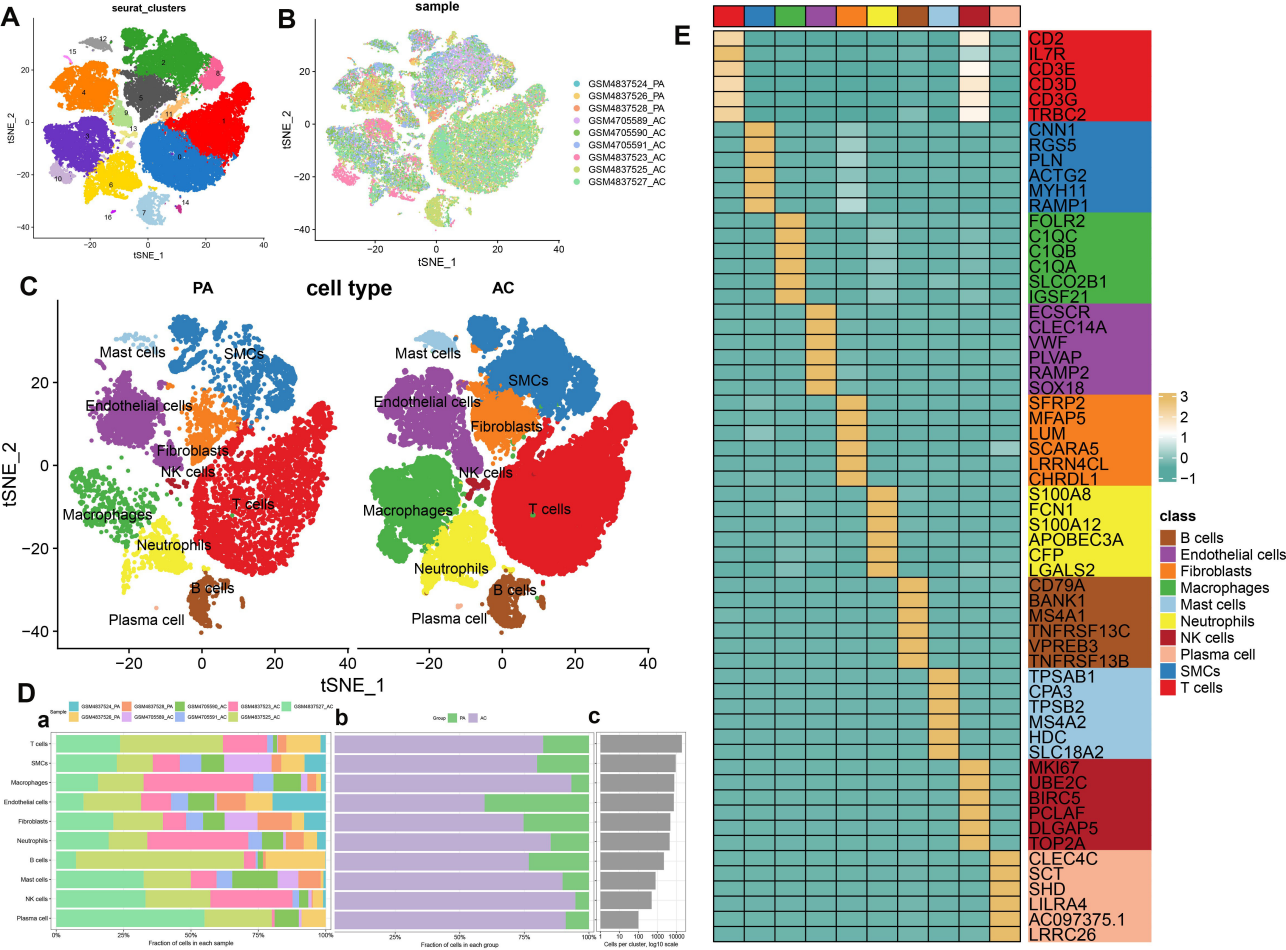
scRNA-seq cell annotation. **(A)** The UMAP plot displays the distribution of the cell clusters of the combinatorial dataset. **(B)**The UMAP plot displays the distribution of the cell clusters of the two types of atherosclerotic plaque. **(C)** The UMAP plot displays the distribution of the cell types of two types of atherosclerotic plaque. **(D)** Cell type fractions of the two types of atherosclerotic plaque. **(E)** A heatmap displayed the distribution of the top 6 differentially expressed genes specific to different cell subtypes.

scRNA-seq analysis was utilized to meticulously characterize the immune cell landscape within atherosclerotic plaques. Following quality control, we identified 55,981 high-quality cells, comprising 11,178 from proximal adjacent (PA) plaque samples and 44,803 from atherosclerotic core (AC) plaque samples, as eligible for subsequent analysis. The distribution of cell clusters across the combined dataset is illustrated in **Figure 1A**, while the cell cluster distribution for the AC and PA groups is depicted in **Figure 1B**. Subsequently, we confirmed 10 immune cell subtypes within these two groups (**Figure 1C**), including T cells (n=19,178) expressing CD3D, smooth muscle cells (SMCs, n=9,122) marked by MYH11, macrophages (n=7,681) identified by FBLN1, endothelial cells (n=7,276) characterized by S100A8, fibroblasts (n=4,767) associated with NKG7, neutrophils (n=4,382) positive for C1QC, B cells (n=2,185) denoted by VWF, mast cells (n=795) signified by CD79A, natural killer (NK) cells (n=495) distinguished by TPSAP1, and plasma cells (n=100) labeled with SDC1 (**Supplementary Figure S1**). The proportion of each cell type within the two groups is presented in **Figure 1D**. The proportion of each cell type within the two groups is presented in **Figure 1D**. The fraction of these ten cell types in each dataset was presented in **Figure 1Da**. In comparison to the PA group, the AC group exhibited higher proportions of most cell types, particularly macrophages and NK cells, whereas epithelial cells were less prevalent (**Figure 1Db**). The absolute numbers of these cells are illustrated in **Figure 1Dc**. These findings reveal a higher enrichment of all immune cell types in the AC group, suggesting that AC patients may experience a more robust immune response compared to PA patients throughout the disease. Additionally, the top six characteristic genes for each cell type are shown in **Figure 1E**. Specifically, the distinguishing genes for T cells are CD2, IL7R, CD3E, CD3D, CD3G, and TRBC2; for SMCs, they are CNN1, RGS5, PLN, ACTG2, MYH11, and RAMP1; for macrophages, FOLR2, C1QC, C1QB, C1QA, SLCO2B1, and IGSF21; for endothelial cells, ECSCR, CLEC14A, VWF, PLVAP, RAMP2, and SOX18; for fibroblasts, SFRP2, MFAP5, LUM, SCARA5, LRRN4CL, and CHRDL1; for neutrophils, S100A8, FCN1, S100A12, APOBEC3A, CFP, and LGALS2; for B cells, CD79A, BANK1, MS4A1, TNFRSF13C, VPREB3, and TNFRSF13B; for mast cells, TPSAB1, CPA3, TPSB2, MS4A2, HDC, and SLC18A2; for NK cells, MKI67, UBE2C, BIRC5, PCLAF, DLGAP5, and TOP2A; and for plasma cells, CLEC4C, SCT, SHD, LILRA4, AC097375.1, and LRRC26. And the enrichment of functional pathways in immune cells is detailed in **Supplementary Figure S2A**. The AC group exhibited elevated scores in nearly all evaluated phenotypes (angiogenesis, ferroptosis, phagocytosis, autophagy, and lysosome function), whereas the PA group demonstrated heightened scores exclusively in cholesterol efflux (**Supplementary Figure S2B**).

### Intercellular communication analysis

**Figure 2:**
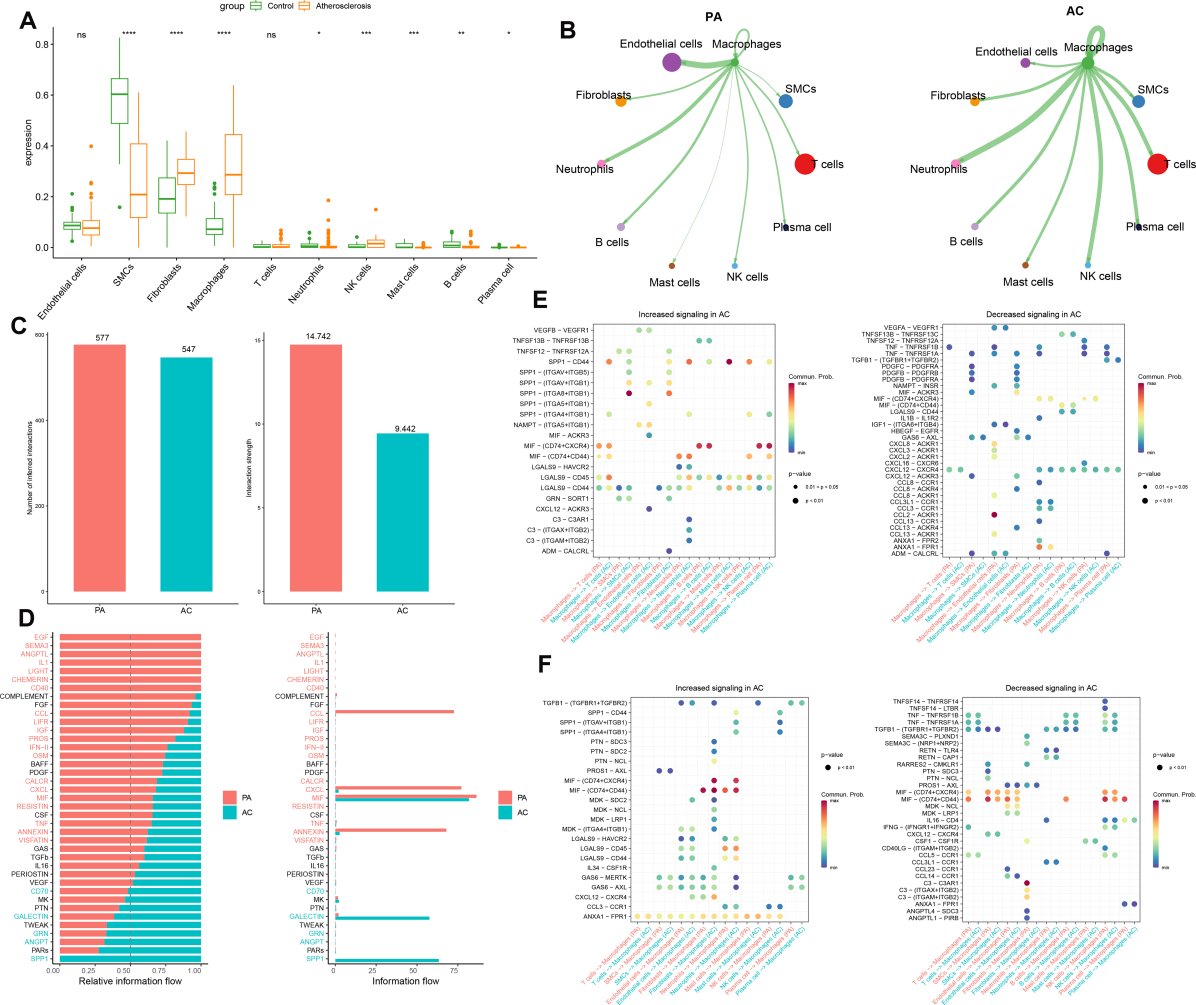
Intercellular communication analysis. **(A)** The cell abundance in the bulk transcriptome dataset GSE100927. **(B)**The strength of interaction between macrophages and other cells in the GSE100927 dataset is depicted graphically. A thicker line represents a stronger interaction, while a larger round dot indicates a larger number of interactions. **(C)** Enumeration and determination of the interaction strength of macrophages in both groups. **(D)** Variations in the intercellular signaling networks between the two groups. **(E)** The macrophages’ functions as a ligand to other immune cells in the two groups are evaluated. **(F)** The macrophages function as receptors to other immune cells in the two groups and are evaluated *p < 0.05, **p < 0.01, ***p < 0.001, ****p < 0.0001. ns means “no significant”.

In this study, we employed the MuSiC algorithm to estimate the distribution of cell subpopulations within the GSE100927 bulk transcriptome dataset by referencing the single-cell data. We conducted intercellular communication analysis between macrophages and other cells within each group (**Figure 2A**). In the PA group, macrophages demonstrated substantial interaction strength with endothelial cells, and neutrophils. Endothelial cells and T cells exhibited a considerable number of interactions with macrophages in the PA group. Additionally, T cells and SMCs exhibited substantial interactions with macrophages in the PA group. Neutrophils, NK cells, and other macrophages demonstrated significant interaction strengths with macrophages (**Figure 2B**). The strength and quantity of macrophage interactions with other cells were compared between the two groups. A higher interaction strength and a greater number of interactions with other cell types were exhibited by macrophages in the PA group compared to the AC group (**Figure 2C**). These findings indicate that immune cells, specifically macrophages, might play a pivotal role in the pathogenesis of PA and AC. Subsequently, we identified distinct signaling pathways linked to macrophages in both groups by analyzing the differences in interaction strengths. In the PA group, pathways such as EGF, SEMA3, ANGPTL, IL-1, LIGHT, CHEMERIN, CD40, CCL, LIFR, IGF, PROS, IFN-II, OSM, CALCR, CXCL, MIF, RESISTIN, TNF, ANNEXIN, and VISFATIN showed elevated activity, with CCL, CXCL, MIF, and ANNEXIN being notably more active than others. In contrast, in the AC group, CD70, GALECTIN, GRN, ANGPT, and SPP1 pathways were active, with SPP1 exclusively so (**Figure 2D**). Intriguingly, MIF exhibited significant activation in both groups. We further investigated key ligand-receptor pairs between macrophages and other cell types (**Figure 2E**). In the PA group, macrophages as ligand cells upregulated interactions such as SPP1-CD44, SPP1-(ITGA8+ITGB1), MIF-(CD74+CXCR4), MIF-(CD74+CD44), and LGAS9-CD45/CD44 while downregulating TNF-TNFRSF1B/TNFRSF1A and CXCL12-CXCR4. Conversely, as receptor cells in the AC group, macrophages enhanced ANX1-FPR1, MIF-(CD74+CD44), GAS6-MERTK/AXL, and LGAS9-CD45/CD44 pathways while inhibiting TGFB1-(TGFBR1+TGFBR2), MIF-(CD74+CXCR4), and MIF-(CD74+CD44) interactions (**Figure 2F**). In this section, we found macrophages in the PA group exhibited stronger and more numerous interactions with endothelial cells, fibroblasts, neutrophils, and B cells compared to those in the AC group. Key signaling pathways in PA included notably active CCL, CXCL, MIF, and ANNEXIN, whereas the AC group showed exclusive activity in pathways like SPP1. Both groups displayed significant MIF pathway activation. Ligand-receptor pair analyses highlighted varied regulatory mechanisms across groups, underscoring macrophages’ critical role in PA and AC pathogenesis.

### The analysis of macrophage heterogeneity in AC

**Figure 3:**
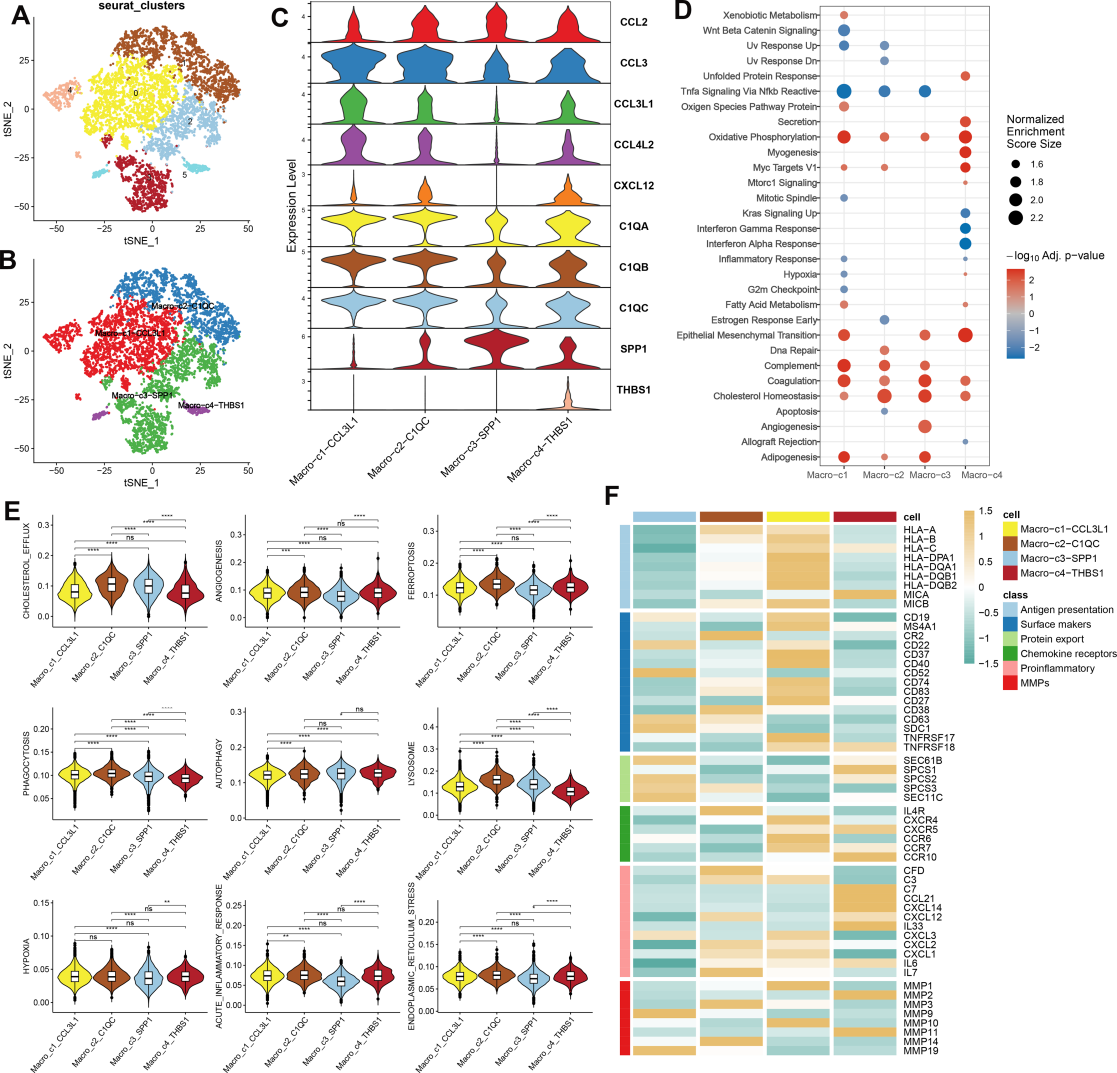
The analysis of macrophage heterogeneity in AC **(A)** The UMAP plot displays the distribution of the cell subclusters of Macrophages in a combined dataset. **(B)**The UMAP plot displays the distribution of the cell subtypes of macrophages in a combined dataset. **(C)** Expression level of marker genes of each subtype of macrophage in AC. **(D)** Functional enrichment analysis subtypes of macrophage in AC. **(E)** Classic phenotypic scores of each macrophage subtype. **(F)** Immune modulators of each macrophage subtype.

A detailed characterization of macrophages within the combined dataset was conducted, initially resulting in the identification of six distinct subclusters (**Figure 3A**). Subsequently, based on the expression of key marker genes, these macrophages were classified into four subtypes: Macro_C1_CCL3L1, Macro_C2_C1QC, Macro_C3_SPP1, and Macro_C4_THBS1 (**Figures 3B, C**). Functional enrichment analysis revealed distinct biological processes associated with each subtype: Macro_C1 was predominantly involved in oxidative phosphorylation, epithelial-mesenchymal transition, complement activation, coagulation, and adipogenesis. Macro_C2 was associated with cholesterol homeostasis and the complement and coagulation pathways. In contrast, Macro_C3 was linked to adipogenesis, angiogenesis, coagulation, and cholesterol homeostasis, while Macro_C4 was involved in oxidative phosphorylation, epithelial-mesenchymal transition, myogenesis, and Myc targets V1 (**Figure 3D**). Additionally, we calculated classic phenotypic scores for each macrophage subtype (**Figure 3E**) and assessed the variability in immune modulators to elucidate the distinctions among the subtypes. Significantly higher levels of immune genes related to antigen presentation, chemokine receptors, and matrix metallopeptidases (MMPs) characterized Macro_C1, while Macro_C2 was noted for elevated immune genes pertinent to surface markers, chemokine receptors, proinflammatory responses, and MMPs. Macro_C3 displayed a high expression of surface marker and protein export-related immune genes alongside notable MMP activity. Finally, Macro_C4 was distinguished by its upregulation of genes involved in proinflammatory responses (**Figure 3F**).

### Development trajectory of macrophage subpopulations

**Figure 4:**
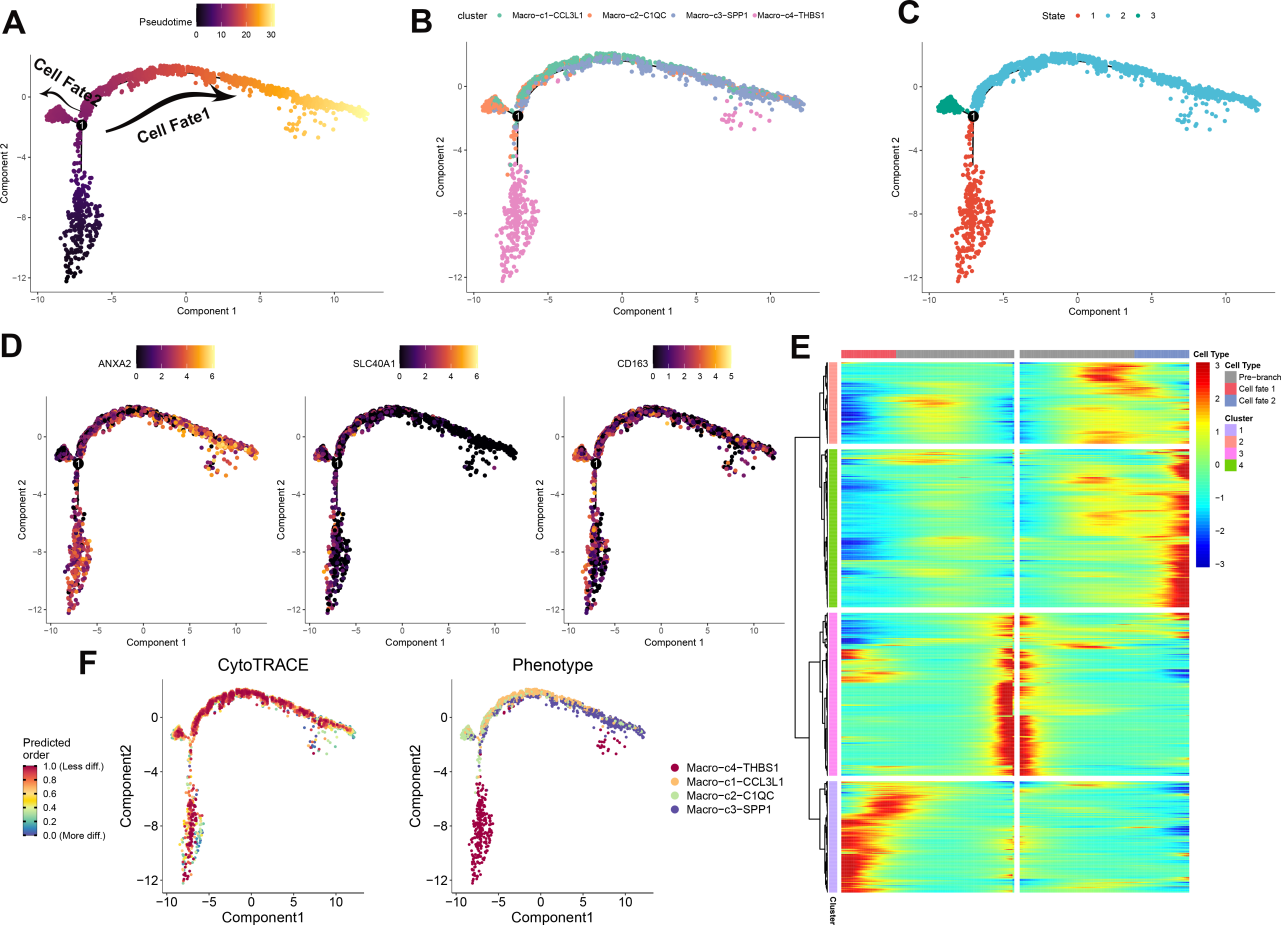
Development trajectory of macrophage subpopulations. The trajectory is depicted using color coding based on pseudotime **(A)**, cell types **(B)**, and states **(C)**. To illustrate the progression of pseudotime, scatter plots **(D)** show the expression levels of specific genes in various cell states. The intensity of color corresponds to the normalized expression of each gene. Additionally, the development pathway of macrophages is represented by assigning colors based on CytoTRACE scores and Phenotype **(F)**. Representative findings highlight the lineage-specific patterns of gene expression during the transformation of cell fate **(E)**.

To elucidate the immune dynamics further, an analysis of pseudotime developmental trajectory was conducted on macrophages to establish the optimal curve representing cell development or differentiation. This analysis inferred the lineage structure of macrophages within the milieu of atherosclerotic plaques. It portrayed a primary developmental trajectory in which macrophages differentiate into two distinct fates over time (**Figure 4A**). Examination of macrophage subtypes revealed that Macro_C3_SPP1 macrophages were situated at one extremity, in contrast to Macro_C2_C1QC macrophages. Meanwhile, Macro_C4_THBS1 and Macro_C1_CCL3L1 macrophages were predominantly centralized, suggesting that Macro_C3_SPP1 macrophages represent a terminal differentiation state (**Figure 4B**). Progression through pseudotime indicated that Macro_C1_CCL3L1 and Macro_C3_SPP1 populations differentiated along the same branch, whereas Macro_C2_C1QC and Macro_C4_THBS1 formed two separate branches, reflecting their inherent heterogeneity (**Figure 4C**). Additionally, the analysis verified differentially expressed genes (ANXA2, SLC40A1, and CD163) along this trajectory. ANXA2 was highly expressed in both C3 and C4 subtypes, SLC40A1 was predominantly expressed in C2, and CD163 was mainly present in C3 and C4 (**Figure 4D**). According to CytoTRACE, Macro_C4_THBS1 macrophages exhibited a higher differentiation potential compared to the other three subtypes, which had lower potential (**Figure 4F**). The abundance of cell state in PA and AC group was presented in **Supplementary Figure S3A, B**. The phenotypes of these four subtypes are presented, with C4 comprising the broadest array of phenotypes and C3 the narrowest (**Figure 4F**; **Supplementary Figure S3C**). The gene expression patterns dependent on lineage along the shifting cell fates were visualized in **Figure 4E**.

### Identification of characteristic genes

**Figure 5:**
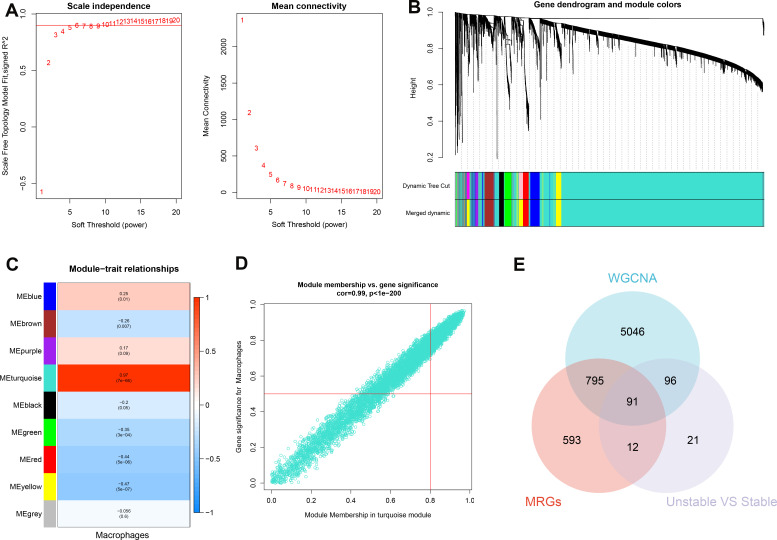
Identification of characteristic genes. **(A)** To compute adjacency in the weighted gene co-expression network analysis (WGCNA), an optimal soft threshold is determined. **(B)** The co-expression modules are clustered, and the resulting dendrogram is displayed. **(C)** The modules that show the strongest correlation with Macrophages are analyzed in the WGCNA. **(D)** The turquoise module is highlighted, and a scatter plot shows the membership of genes in this module. **(E)** The interaction among the characteristic genes identified from the WGCNA, pseudotime analysis (MRGs), and hallmark genes in E-MTAB-2055 (unstable vs stable plaques) is examined.

The WGCNA algorithm was utilized to establish a gene co-expression network for GSE100927. An optimal soft-thresholding power β of 6 facilitated the application of a hierarchical clustering algorithm to the sample dataset, culminating in the elucidation of nine distinct gene co-expression modules differentiated by color in the clustering dendrogram as depicted in **Figures 5A-C**. Notably, the turquoise module demonstrated a robust correlation (R=0.97) with macrophages, yielding 6028 genes for further investigation (**Figure 5D**). In addition, we observed a significant positive relationship between the turquoise module and its associated genes. Pseudotime analysis yielded 1491 module-related genes, among which 220 hallmark genes were validated by comparing stable and unstable plaque samples from E-MTAB-2055. Subsequently, by intersecting data from WGCNA, pseudotime analysis, and the hallmark genes derived from E-MTAB-2055, we identified 91 distinctive genes, as illustrated in **Figure 5E**.

Enrichment analyses were subsequently conducted to elucidate the potential biological functions of these 91 genes. Gene Ontology (GO) analysis indicated a broad involvement of these genes in biological processes (BP) such as neutrophil activation, intracavitary enzyme and cytokine secretion (cellular component, CC), and protein complex binding (molecular function, MF), detailed in **Supplementary Figure S4A**. Furthermore, Kyoto Encyclopedia of Genes and Genomes (KEGG) pathway analysis revealed extensive enrichment in the activities and metabolism of lysosomes, cytokines, and cholesterol, as shown in **Supplementary Figure 4SB**.

### Screening of characteristic genes based on machine learning

**Figure 6:**
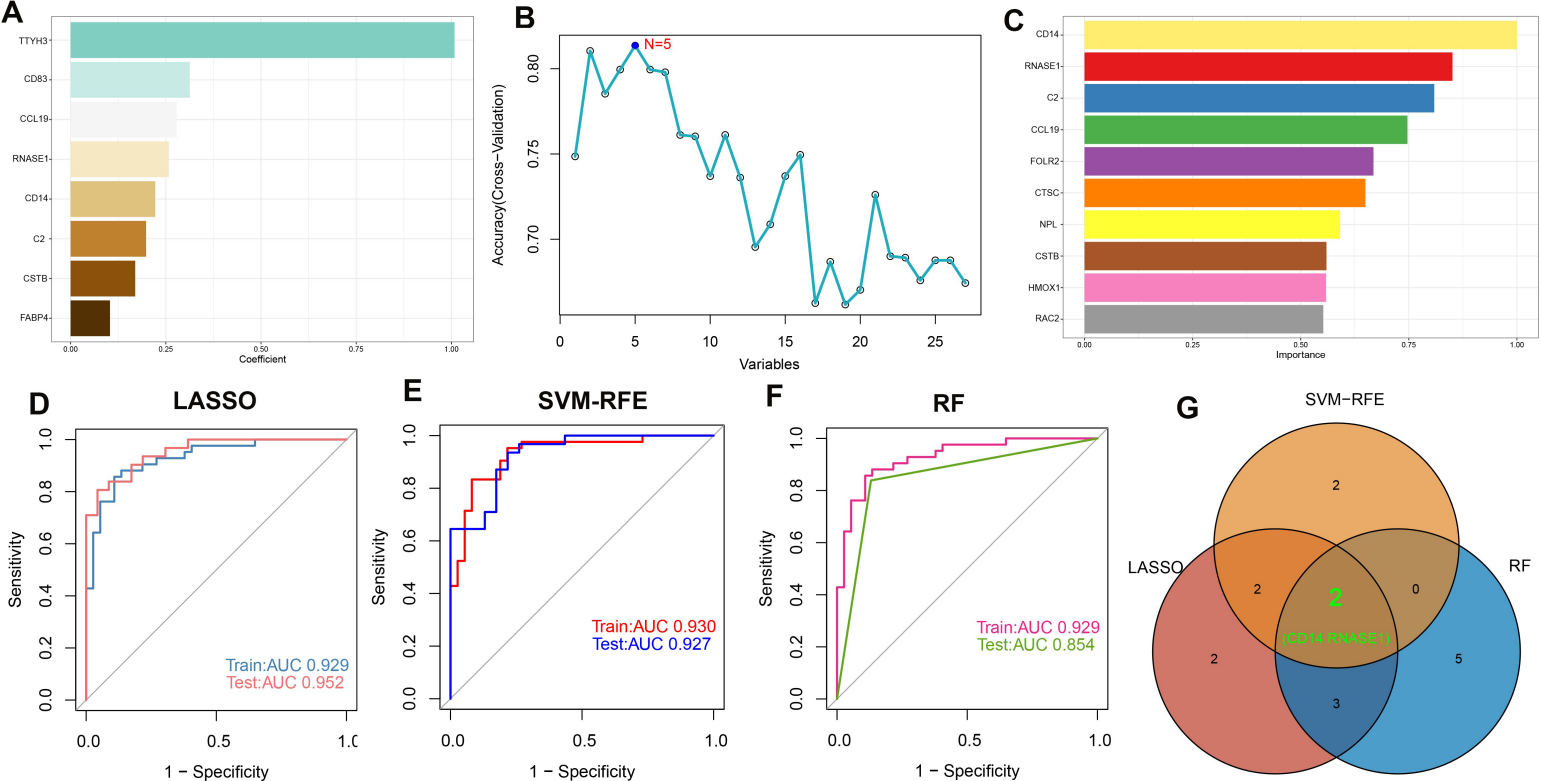
Machine learning-based feature gene selection. **(A)** Variables with non-zero coefficients identified by LASSO. **(B)** In variable selection using SVM, the highest accuracy was achieved when N=5. **(C)** Top 10 variables ranked by importance in the RF model. The ROC curves and AUC values of models are built based on their characteristic genes constructed by the LASSO **(D)**, SVM-RFE **(E)**, and RF **(F)** algorithms in the training and testing cohorts. **(G)** Venn plot of feature genes selected by LASSO, SVM-RFE, and RF.

To identify potential biomarkers for atherosclerosis diagnosis, we utilized machine learning algorithms to pinpoint salient features. Before merging, we examined the distribution of the three datasets and assessed the uniformity of data following background calibration, as depicted in **Supplementary Figures S5A, B**. This analysis confirmed the successful mitigation of batch effects among the datasets. The combined datasets, which included both the training and validation sets, then underwent further processing. Initially, we identified genes with non-zero coefficients—TTYH3, CD83, CCL19, RNASE1, CD14, C2, CSTB, and FABP4—using the Least Absolute Shrinkage and Selection Operator (LASSO) technique (refer to **Figure 6A**; **Supplementary Figures S5C, D**). Subsequently, the Boruta algorithm pinpointed 27 significant variables (shown in **Supplementary Figure S5E**). These variables were individually assessed within Support Vector Machine (SVM) and Random Forest (RF) models to refine variable selection (**Figures 6B, C**). In the training set, Receiver Operating Characteristic (ROC) curve analysis revealed that the LASSO model possessed an Area Under the Curve (AUC) of 0.929, while in the test set, the AUC was 0.952, demonstrating superb predictive capacity for atherosclerosis (**Figure 6D**). The SVM with Recursive Feature Elimination (SVM-RFE) and RF models were similarly effective, showing high predictive accuracy with robust AUC values for both the training and test sets (**Figures 6E, F**). Ultimately, a Venn diagram pinpointed CD14 and RNASE1 as the distinctive characteristic genes for atherosclerosis, derived from the intersected findings of the three modeling techniques (**Figures 6G**).

### Establishing and validating a diagnostic riskScore model

**Figure 7:**
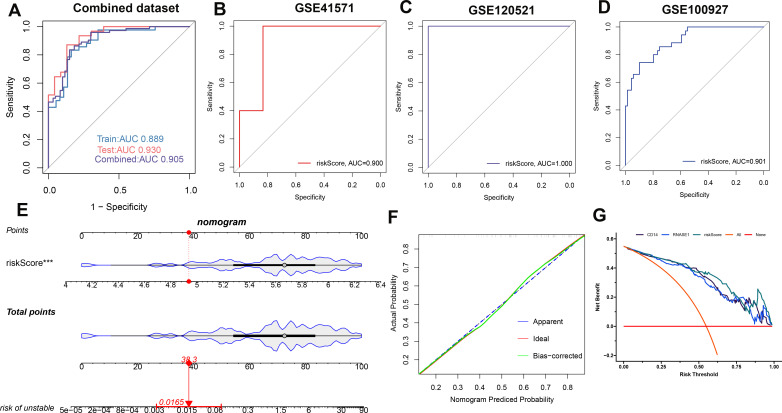
Establishing and validating a diagnostic riskScore model. To assess the model’s performance, ROC curves were employed for the combined dataset **(A)**, GSE41571 **(B)**, GSE120521 **(C)**, and GSE100527 **(D)**. Additionally, the riskScore model in the combined dataset is presented as a nomogram **(E)**. To ensure its stability, a calibration curve was utilized **(F)**. Furthermore, the clinical benefit of the nomogram is illustrated through DCA analysis **(G)**.

A risk score model was developed utilizing the formula derived from the respective least absolute shrinkage and selection operator (LASSO) model coefficients of two distinct genes: Risk Score = (-0.22231781 × Expression of CD14) + (0.257829399 × Expression of RNASE1). Receiver operating characteristic (ROC) analysis across the combined dataset and the individual datasets (GSE41571, GSE120521, and GSE10097) yielded a high area under the curve (AUC) values exceeding 0.8. This result demonstrates the diagnostic model’s high accuracy, as indicated by the risk score (**Figures 7A-D**). Furthermore, we constructed a nomogram for predicting atherosclerosis based on the risk score model. We confirmed the nomogram’s stability using a calibration curve (**Figures 7E, F**). Additionally, decision curve analysis (DCA) for the nomogram revealed potential clinical benefits for patients diagnosed with atherosclerosis (**Figures 7G**).

### Molecular characteristics and functional annotation of the riskScore model in atherosclerosis

**Figure 8:**
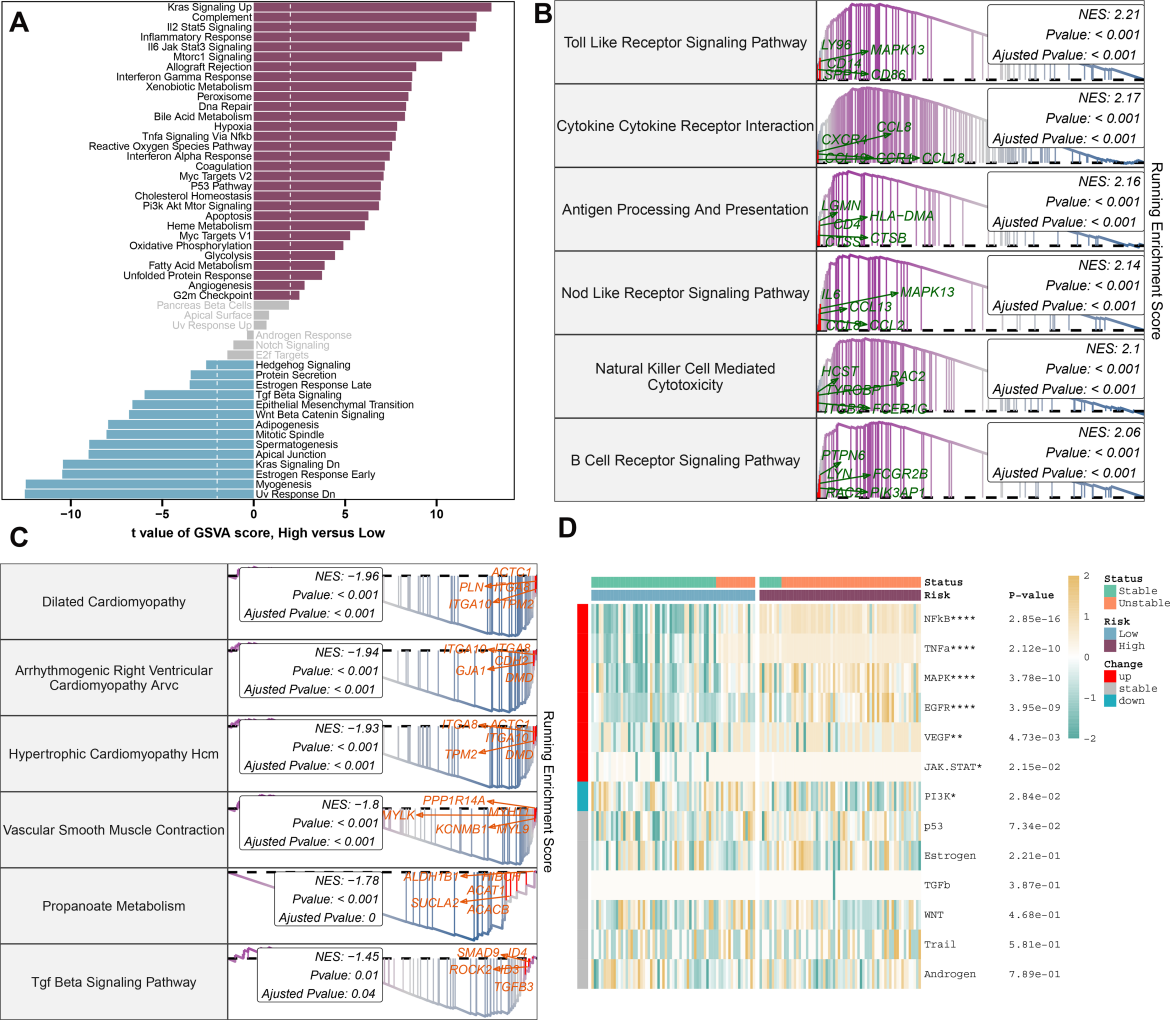
Molecular characteristics and functional annotation of the riskScore model in atherosclerosis. **(A)** Investigation of the variances in biological function among high and low-risk groups is conducted. **(B)** The top 6 pathways showing up-regulation in the high-risk group are identified. **(C)** The top 6 pathways that display down-regulation in the high-risk group are identified. **(D)** A heatmap illustrating the disparities in pathogenic pathways between AS patients at low and high risk is exhibited. The patient annotations signify their stable and unstable status. Statistical significance levels: *p < 0.05, **p < 0.01, ***p < 0.001, ****p < 0.0001.

To better understand the mechanisms underlying atherosclerosis, we investigated the feature genes of both high- and low-risk groups. We identified 462 genes that were downregulated and 653 genes that were upregulated, isolating the top 5 genes in each category: RNASE1, MFNG, C1QA, HCK, and SLAMF8 for upregulated genes, and SHROOM3, SVIL, VCL, NEXN, and LMOD1 for downregulated genes (**Supplementary Figures S6A-C**). Subsequent annotation of the associated biological functions, and pathway enrichment analyses using GSVA and GSEA, revealed distinct profiles between the groups. The low-risk group showed significant enrichment in biological functions related to hormone secretion and cellular development, including UV response down, myogenesis, early estrogen response, KRAS signaling down, apical junction, spermatogenesis, mitotic spindle, adipogenesis, Wnt beta-catenin signaling, late estrogen response, and protein secretion. In contrast, the high-risk group’s functions are predominantly related to immunoinflammatory responses and cholesterol metabolism (**Figure 8A**). We further characterized the top 6 upregulated pathways (Toll-like receptor signaling, cytokine-cytokine receptor interaction, antigen processing and presentation, NOD-like receptor signaling, natural killer cell-mediated cytotoxicity, and B cell receptor signaling pathway) and the top 6 downregulated pathways (dilated cardiomyopathy, arrhythmogenic right ventricular cardiomyopathy ARVC, hypertrophic cardiomyopathy HCM, vascular smooth muscle contraction, propanoate metabolism, TGF-beta signaling pathway) in the high-risk group using GSEA (**Figures 8B, C**). Moreover, pathogenic pathway activities were markedly different between patients with varying atherosclerosis risk levels. High-risk patients exhibited significant activity in pathways including NF-κB, TNF-α, MAPK, EGFR, VEGF, and JAK-STAT, whereas low-risk patients demonstrated an upregulation of JAK-STAT and downregulation of PI3K pathways (**Figure 8D**).

### Immune characteristics analysis

**Figure 9:**
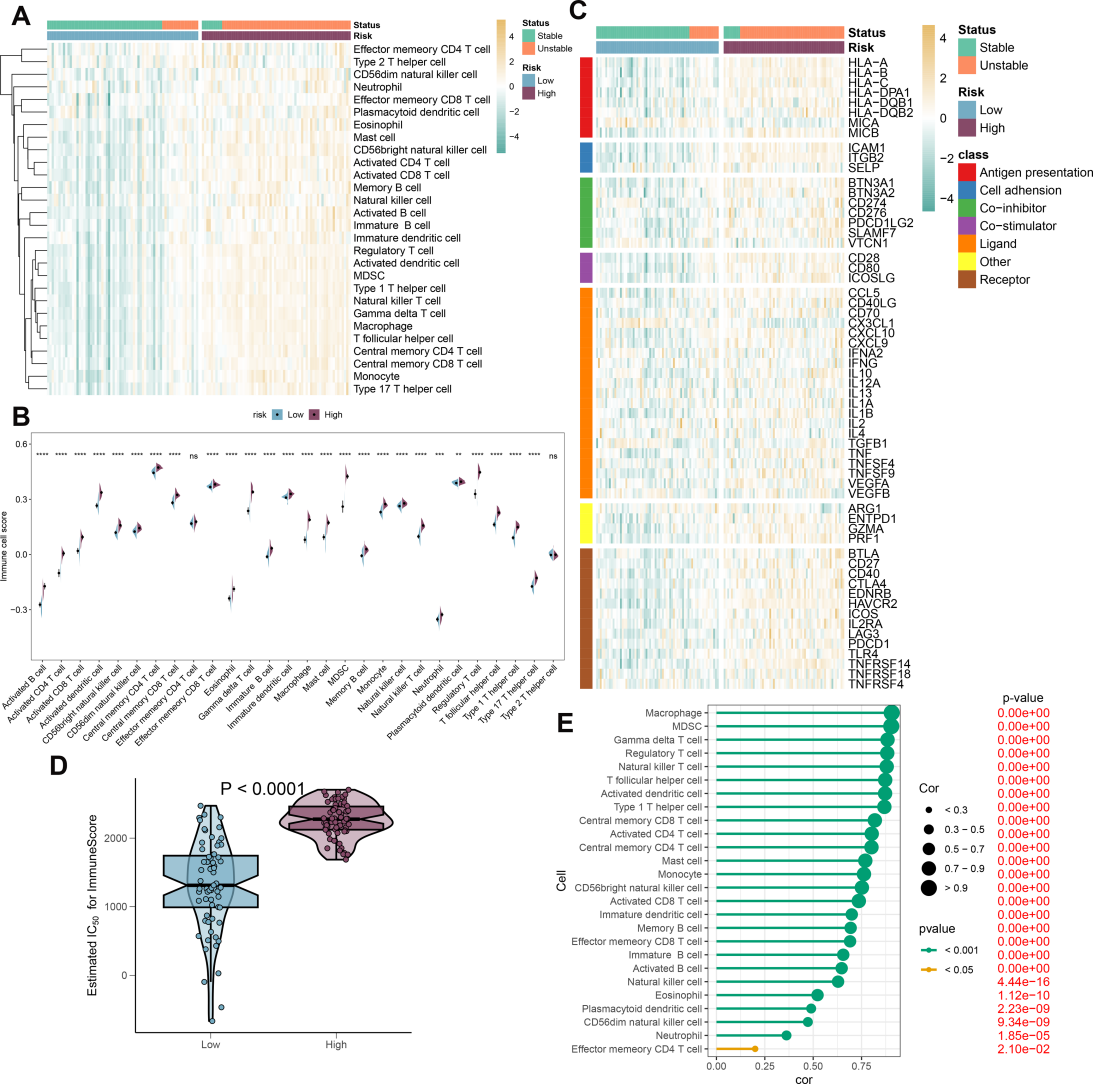
Immunological features of AS patients at low and high risk. **(A)** The heatmap displays the level of infiltration of 28 subtypes of immune cells in the two risk groups. **(B)** Contrasts in immune cell scores are observed between the high- and low-risk categories. **(C)** The heatmap highlights the variances in immune-modulating factors as well as the survival status of patients between the high- and low-risk groups. **(D)** A comparison of the immuneScore is made between the high- and low-risk groups. **(E)** Understanding the relationship between the riskScore and the various subtypes of immune cells is explored. **p < 0.01, ***p < 0.001, ****p < 0.0001. ns means “no significant”.

To elucidate the immune cell landscape in patient populations stratified by risk and stability status, we first analyzed 28 distinct immune cell subtypes using the ssGSEA algorithm and compared their infiltration between high-risk and low-risk groups (**Figure 9A**). Generally, the high-risk group exhibited increased infiltration across the majority of immune cell subtypes. Furthermore, samples from the high-risk group were predominantly associated with an unstable condition. Aligning with prior findings, individuals in the high-risk category consistently demonstrated elevated immune cell scores for nearly all cell types (**Figure 9B**). Additionally, we evaluated the differential expression of immune modulators between the high- and low-risk cohorts, whether stable or unstable, to provide deeper insight into the immune landscape pertinent to atherosclerosis pathology (**Figure 9C**). Predominantly, genes involved in antigen presentation (HLA-A, B, C, DPA1, DQB1, MICB), cell adhesion (ICAM1, ITGB2, SELP), immune checkpoints (BTN3A1, BTN3A2, CD276, PDCD1LG2, SLAMF7), co-stimulation (CD28, CD80, ICOSLG), ligand production (CCL5, CD40LG, CD70, CXCL10, CXCL9, IL10, IL12A, IL1B, TGFB1, TNF, TNFSF9, VEGFA), receptor expression (BTLA, CD27, CD40, CTLA4, EDNRB, HAVCR2, ICOS, IL2RA, LAG3, PDCD1, TRL4, TNFRSF14, TNFRSF18), among others (ENTPD1, GZMA, PRF1), were substantially increased in the high-risk group. By contrast, the low-risk group primarily showed elevated levels of antigen presentation (MICA) and ligands (CX3CL1) expression (**Supplementary Figure S7A-G**). Immune scores were quantitatively compared across risk profiles, reflecting a comprehensive evaluation of immunological characteristics, with the high-risk group presenting with higher immune scores than their counterparts (**Figure 9D**). In addition, correlation analysis linked higher risk scores with greater immune cell type abundance and infiltration (**Figure 9E**). In conclusion, the findings from this section confirm an intensified immune cell activity and response in high-risk atherosclerosis patients.

### Validation of characteristic genes

**Figure 10:**
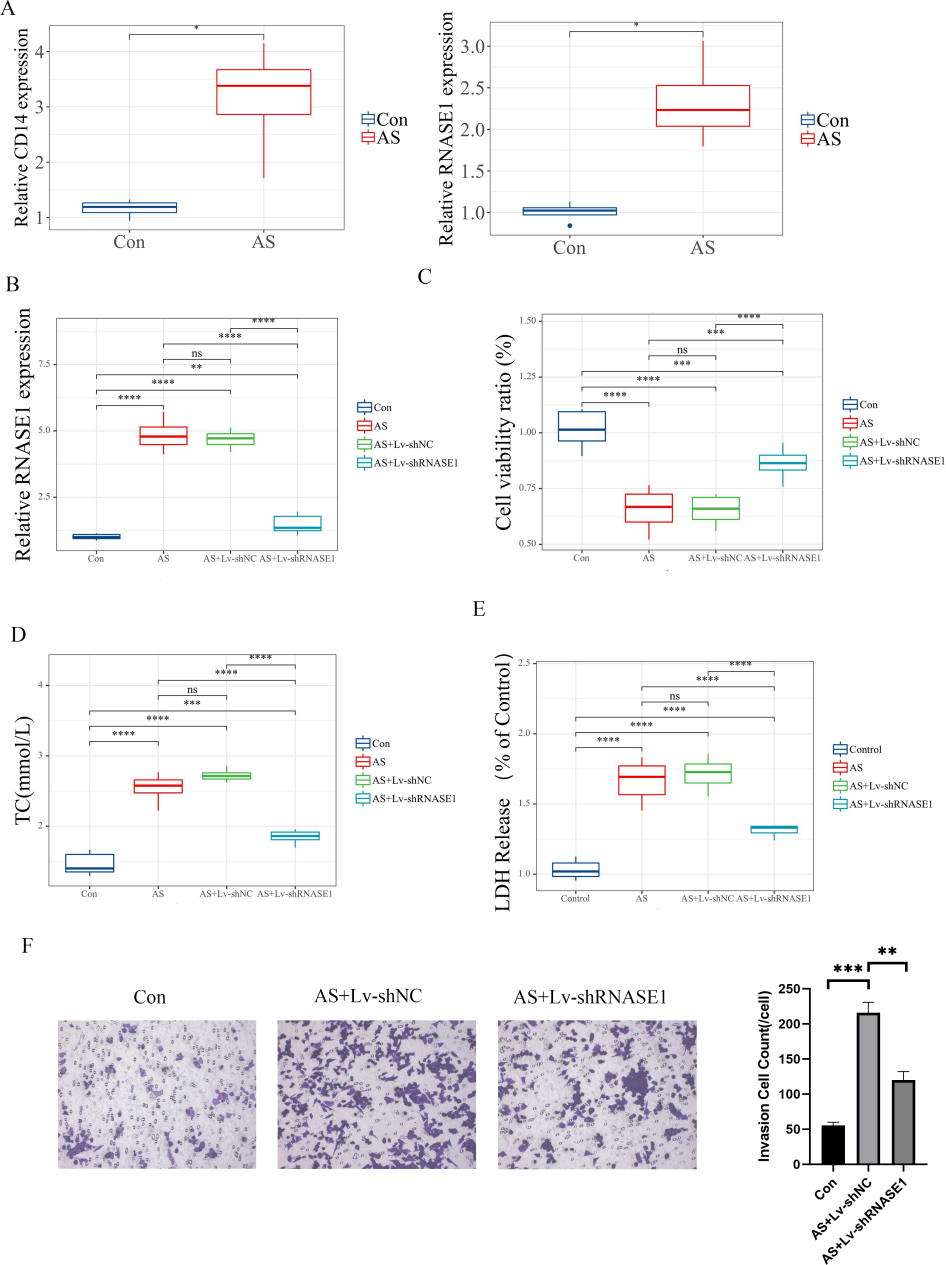
*In vitro* validation of distinct genes. **(A)** The relative expression levels of these characteristic genes were assessed in different groups. **(B)** The analysis specifically focused on RNASE1 expression in various groups, including the control, AS, AS+Lv-shNC, and AS+Lv-shRNASE1 groups. **(C)** Additionally, the viability of cells was measured for each group. **(D, E)** The levels of TC and LDH release were also evaluated in each group. **(F)** Transwell assay and quantitative analysis of each group. Statistical significance is indicated by *p < 0.05, **p < 0.01, ***p < 0.001, ****p < 0.001. ns means “no significant”.

Furthermore, we developed both *in vivo* and *in vitro* AS models to validate the role of characteristic genes. *In vitro*, Expression analyses revealed differential levels of CD14 and RNASE1 in AS (**Figure 10A**). Quantitative RT-PCR was utilized to evaluate the efficiency of the knockdown models. In the AS+Lv-shRNASE1 group, RNASE1 expression was reduced to approximately one-quarter of that observed in the AS+Lv-shNC group (**Figure 10B**).

**Figure 11:**
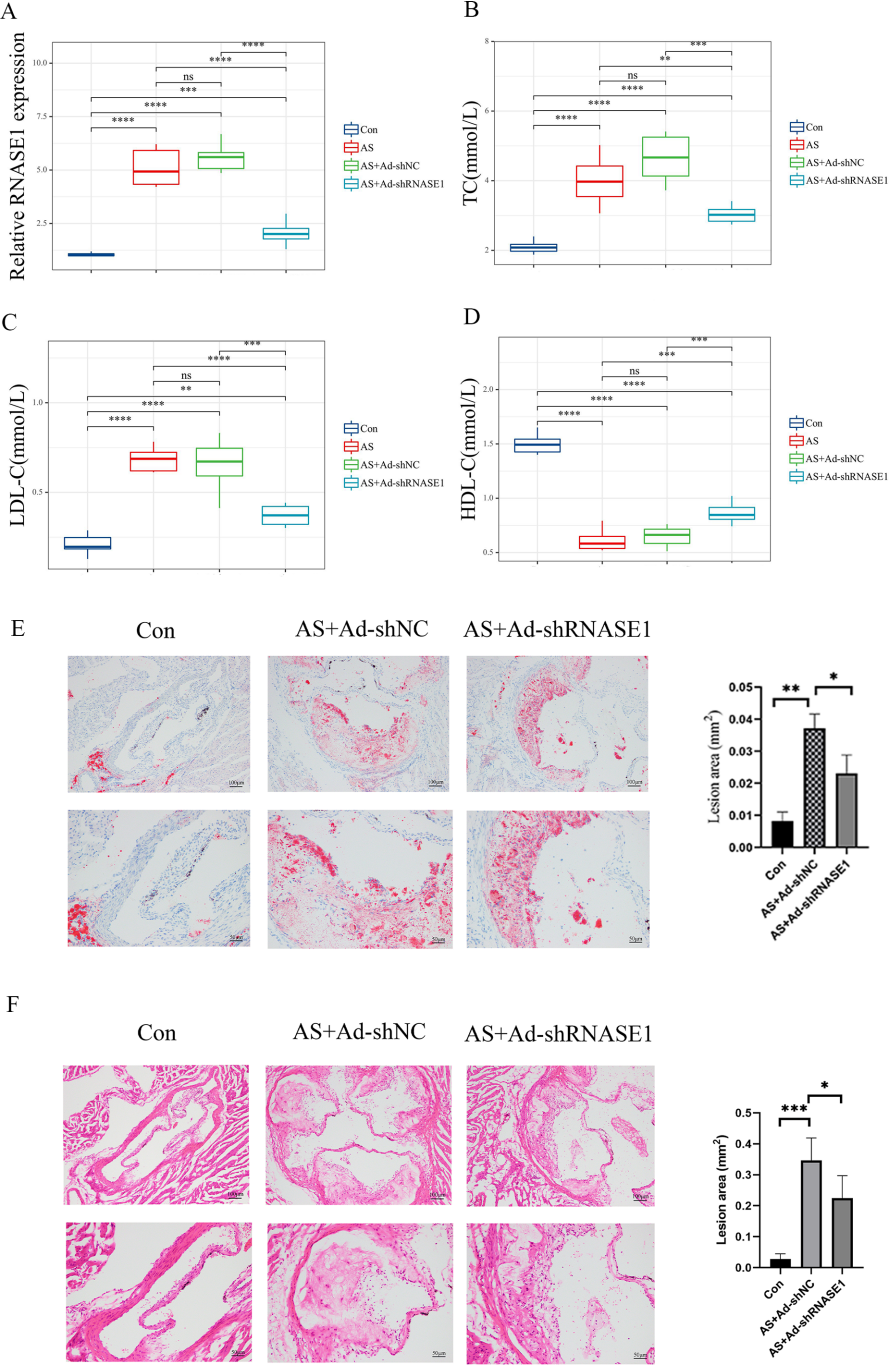
Verification of characteristic genes *in vivo* **(A)** Relative expression levels of RNASE1 in the control group, AS group, As+Ad-LvNC group, and AS+Ad-shRNASE1 group. **(B)** Levels of TC in each group. **(C)** Levels of LDL-C in each group. **(D)** Levels of HDL-C in each group. **(E)** Aortas of rats in the Control, AS+Ad-shNC, and AS+Ad-shRNASE1 group stained with oil red O. **(F)** Aortas of rats in the Control, AS+Ad-shNC, and AS+Ad-shRNASE1 group stained with hematoxylin and eosin. *p < 0.05, **p < 0.01, ***p < 0.001, ****p < 0.001. n=3 in each group.

*In vivo*, a similar reduction was noted in the AS+Ad-shRNASE1 group, with a threefold decrease in RNASE1 expression compared to the AS+Ad-shNC group (**Figure 11A**). These results confirmed the effective knockdown in both *in vivo* and *in vitro* settings. *In vitro*, RNASE1 knockdown resulted in enhanced cell viability and a reduction in total cholesterol (TC) and lactate dehydrogenase (LDH) release in the AS model (**Figures 10C-E**). The migration intensity of cells in the AS intervention group was significantly higher than that in the control group, suggesting enhanced activation and recruitment of macrophages in the AS model. Furthermore, the silencing of RNASE1 was shown to diminish macrophage migration in this model (**Figure 10F**). Additionally, rats in the AS+Ad-shRNASE1 group showed lower levels of TC and low-density lipoprotein cholesterol (LDL-C) and higher levels of high-density lipoprotein cholesterol (HDL-C) in peripheral blood compared to the AS+Ad-shNC group (**Figures 11B-D**). These findings suggest knocking down RNASE1 may diminish the risk of atherosclerotic plaque formation. Oil Red O staining of the aortas revealed the absence of lipid plaques in the control group, whereas extensive red lipid plaque formation was observed along the thoracic aorta in the AS model. This plaque was remarkably reduced in the aortas of AS+Ad-shNC rats (**Figure 11E**). Additionally, hematoxylin and eosin (HE) staining indicated that the AS-induced pathological changes in the aortas were significantly ameliorated in the AS+Ad-shNC group (**Figure 11F**). Collectively, these findings underscore the pivotal role of RNASE1 in atherosclerosis development, implicating this gene in LDL, HDL, and cholesterol metabolism.

## Discussion

Cardiovascular disease (CVD) accounts for nearly half of all fatalities from non-communicable diseases worldwide, establishing it as the primary cause of death on a global scale (35). While commonly recognized risk factors for coronary artery disease, the most prevalent form of CVD, include hypertension, high cholesterol, age, and genetics, it is now understood that CVDs are chronic inflammatory conditions (36). The accumulation of excess cholesterol within the arterial wall leads to the development of arterial blockages, known as atherosclerosis, creating a state of perpetual inflammation. This inflammatory state causes changes in the surrounding components of the vessel wall, such as the endothelial, smooth muscle, and extracellular matrix, leading to disease progression (37). Macrophages, which can originate from either resident tissues or monocytes, play a crucial role in the advancement and regression of atherosclerotic disease (38, 39). During the initial stage of atherogenesis, the endothelial cells secrete chemokines, which attract macrophages to the arterial wall. This attraction is facilitated by the interaction between chemokines and receptors, as well as the expression of cell adhesion molecules like intercellular adhesion molecule 1 (ICAM1) and vascular cell adhesion molecule 1 (VCAM1) (40). The accumulation of cholesterol in macrophages stimulates inflammatory reactions, which involve the activation of Toll-like receptor (TLR) signaling and NF-κB-mediated activation of the NLRP3 inflammasome. As a result, pro-inflammatory cytokine production increases, further intensifying the chronic inflammatory state in atherosclerosis (41). Diminishing macrophage apoptosis and enhancing their efferocytic activity may constitute an innovative therapeutic approach aimed at mitigating necrotic core formation and vascular calcification, consequently augmenting the stability of atherosclerotic plaques (42). Diverse macrophage populations are present within human atherosclerotic lesions, although much of their functional characteristics remain elusive. A recent study has demonstrated that crosstalk between macrophages and endothelial cells exacerbates atherosclerosis in male mice (43). Macrophage angiotensin-converting enzyme mitigates atherosclerosis by enhancing peroxisome proliferator-activated receptor alpha, thereby substantially altering lipid metabolism (44). Acquiring a comprehensive understanding of the mechanisms that dictate macrophage phenotypes in the context of atherosclerosis may unveil novel therapeutic prospects.

In our research, we conducted scRNA-seq analysis on both proximal adjacent (PA) plaques and the atherosclerotic core (AC) plaques. This analysis revealed a heightened immune cell activity within the AC plaques, designating them as “immune plaques.” Macrophages, in particular, were identified as the predominant cell type within these immune plaques. Furthermore, the study elaborates on the intricate intercellular communication, specifically the ligand-receptor interactions and the associated pathways, between macrophages and other immune cells. Macrophages interacted robustly with endothelial cells, fibroblasts, and others in PA, while exhibiting strong interactions with neutrophils and T cells in AC. This suggests macrophages’ pivotal role in PA and AC pathogenesis. Pathway analysis highlighted active signaling in PA, notably CCL, CXCL, MIF, and ANNEXIN, whereas AC showed distinct activity in pathways like SPP1. Specific ligand-receptor pairs further underscored differential regulatory mechanisms in macrophage interactions between the two groups. In addition, we classified macrophages into 4 subtypes (Macro_C1_CCL3L1, Macro_C2_C1QC, Macro_C3_SPP1, and Macro_C4_THBS1), conducted enrichment analysis, and accessed immune-modulators of the 4 subtypes. Cell trajectory analysis demonstrated that Macro_C3_SPP1 macrophages appeared primarily toward the end of the differentiation trajectory. Differentially expressed genes (ANXA2, SLC40A1, and CD163) along the trajectory were verified. Annexin A2 (ANXA2) is an integral protein within the annexin family, ubiquitously expressed on the surfaces of macrophages, and exhibits a high affinity for calcium and phospholipids, playing a pivotal role in diverse biological processes such as endocytosis, cell-to-cell communication, and plasminogen activation (45). Solute carrier family 40 member 1(SLC40A1) mediates ferroptosis in diseases including diabetes, cardiac dysfunction, and hepatocellular carcinoma (46–48). A recent study has identified SLC40A1 as a pivotal gene implicated in iron metabolism within airway macrophages, specifically in the context of childhood allergic asthma (49). Hemoglobin-haptoglobin receptor CD163 positive macrophages were associated with plaque progression, microvascularity, and a high level of HIF1α and VEGF-A expression in atherosclerosis (50). ANXA2 had high expression levels in C3 and C4, SLC40A1 predominantly expressed in C2 and CD163 mainly expressed in C3 and C4. Investigations into the roles of ANXA2 and SLC40A1 in the pathogenesis of atherosclerosis are notably scarce. Given the established involvement of these proteins in macrophage-associated mechanisms within atherosclerotic processes, they merit more in-depth examination. In this part of the study, we provide a comprehensive delineation of macrophage heterogeneity in arteriosclerotic tissues, outlining their possible functional targets. Additionally, we have characterized the subtypes and phenotypic traits apparent during distinct differentiation phases, revealing putative therapeutic endpoints for the tailored management of arteriosclerosis.

Transcriptome analyses were then used to further screen for signature genes associated with macrophages in atherosclerosis and 91 genes were acquired by interacting genes from WGCNA, pseudotime analysis, and hallmark genes in E-MTAB-2055. Enrichment analyses were performed to further demonstrate the potential function of the 91 genes. Then machine learning algorithms were conducted to predict the characteristic genes and the diagnostic efficiency was confirmed by ROC curve analysis. Based on the distinct characteristic genes (CD14 and RNASE1), a macrophage-related risk riskScore model and nomogram were established with reliable diagnostic performance. Based on the risk scoring, patients were stratified into high-risk and low-risk groups. A subsequent analysis was conducted to examine the differential expression of characteristic genes between these groups. Furthermore, GSVA and GSEA were employed to assess their functional implications. We discovered that the high-risk group exhibited a substantial enrichment of genes associated with immune-inflammatory responses and immune-related pathways (NFK-β, TNF-α, MAPK, EGFR, VEGF, and JAK.STAT). Subsequent immune characteristics analysis further validated our previous findings.

Interestingly, the findings suggest that RNASE1 may be a potential key gene implicated in the involvement of macrophages in the pathogenesis of atherosclerosis. RNASE1 is an essential enzyme for maintaining vascular homeostasis by safeguarding endothelial cells against the damaging effects of extracellular RNA during acute inflammation (51). It is pivotal in averting endothelial dysfunction, a central factor in vascular pathologies like atherosclerosis (52, 53). However, recent research indicated that RNASE1 can act as a protective agent in acute inflammatory settings, yet may contribute to disease progression in chronic inflammation (54–56). We further confirmed the function of RNASE1 in atherosclerotic disease models at both the *in vivo* and *in vitro* levels. Inhibition of RNASE1 in an atherosclerosis macrophage cell line resulted in decreased total cholesterol (TC) and lactate dehydrogenase (LDH) release. *In vivo*, RNASE1 knockdown lowered TC and low-density lipoprotein cholesterol (LDL-C) levels, and increased high-density lipoprotein cholesterol (HDL-C) in the peripheral blood of AS rats. Furthermore, the knockdown of RNASE1 could reduce the migration level of macrophages in the AS model. macrophage migration, recruitment, and activation play pivotal roles in plaque formation, and modulating their activity can have beneficial effects (57). Additionally, RNASE1 suppression led to a reduction in the aortic plaque area in these rats. Our findings highlight RNASE1’s integral role in atherosclerosis progression, implicating the gene in low-density lipoprotein (LDL), high-density lipoprotein (HDL), and cholesterol metabolism. Yet, to elucidate the detailed molecular mechanisms behind RNASE1’s impact on macrophage function in atherosclerosis, further experimental inquiry is crucial.

This study is subject to inherent limitations, primarily due to its retrospective nature and the relatively small sample size drawn from public datasets, highlighting the necessity for validation through prospective studies in diverse institutions. Moreover, the accuracy of the outcomes could be compromised by the reliance on transcriptomic data derived from microarray datasets, which may lack the consistency of data obtained from more controlled experimental conditions. Enhancing the study’s clinical applicability to atherosclerotic patients with varied molecular profiles and risk factors would require the inclusion of a larger participant pool, thus facilitating a more thorough evaluation of prognostic and therapeutic implications.

## Conclusion

The study provides significant insights into the immune-mediated mechanisms underlying atherosclerosis by precisely identifying diverse macrophage subpopulations and elucidating their complex interactions within atherosclerotic plaques. Diagnostic biomarkers, RNASE1, and CD14 have been identified, and a high-precision risk score model has been developed, which are considered valuable tools for clinical diagnosis and patient stratification. Furthermore, the potential of RNASE1 as a therapeutic target has been unveiled, enhancing the current understanding and opening avenues for pioneering treatment strategies for atherosclerosis.

## Data Availability

The raw data supporting the conclusions of this article will be made available by the authors, without undue reservation.
