# Supplementary material for: Immune and inflammatory insights in atherosclerosis: development of a risk prediction model through single-cell and bulk transcriptomic analyses
Source: Front Immunol. 2024 Sep 19;15:1448662. doi: 10.3389/fimmu.2024.1448662 (PMC11446800; doi:10.3389/fimmu.2024.1448662)
Supplement: Supplementary file 1 [file DataSheet1.docx]

**
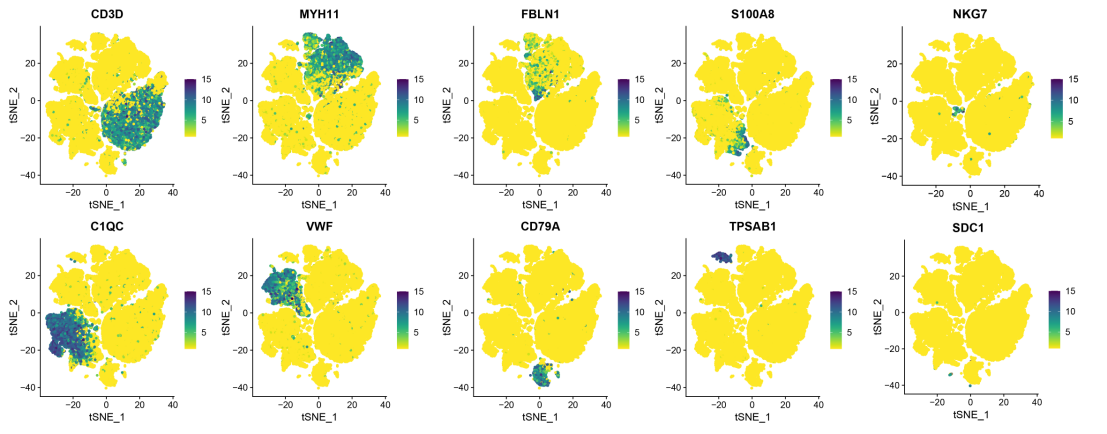
**

**FigureS1 Distinctive marker genes for each cell subtype.**


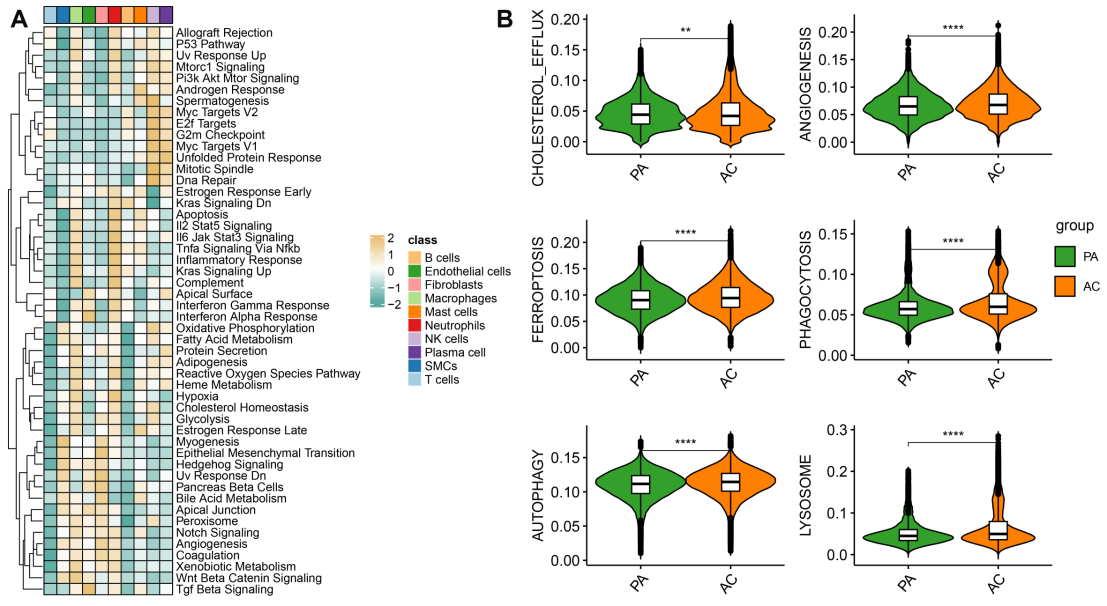


**FigureS2 (A)The enrichment of functional pathways of immune cells in the combintion dateset (B) Classic phenotypic scores in each group.**

**
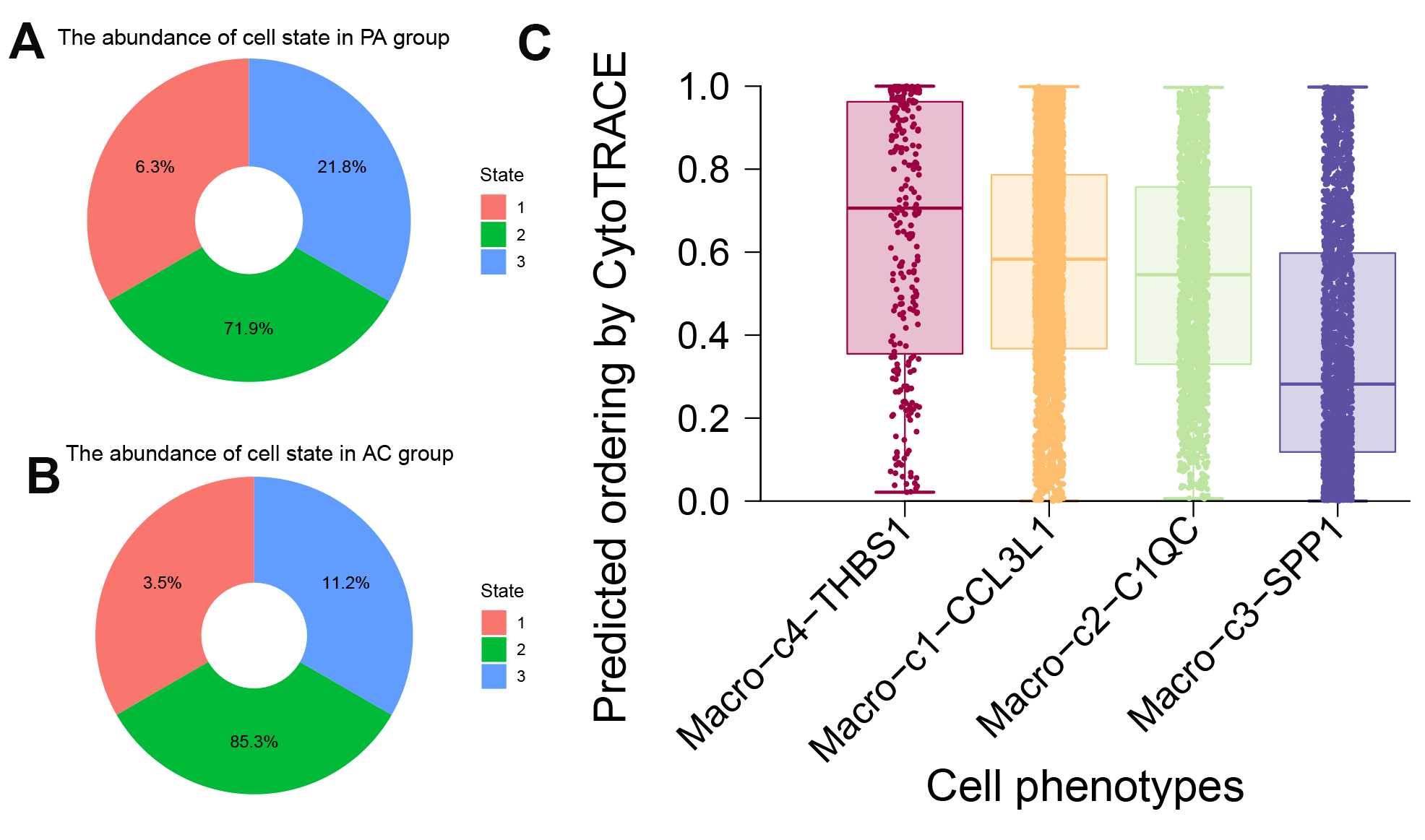
**

**FigureS3 (A,B)** The abundance of cekk state in AC group **(C)**The phenotypes of the four subtypes


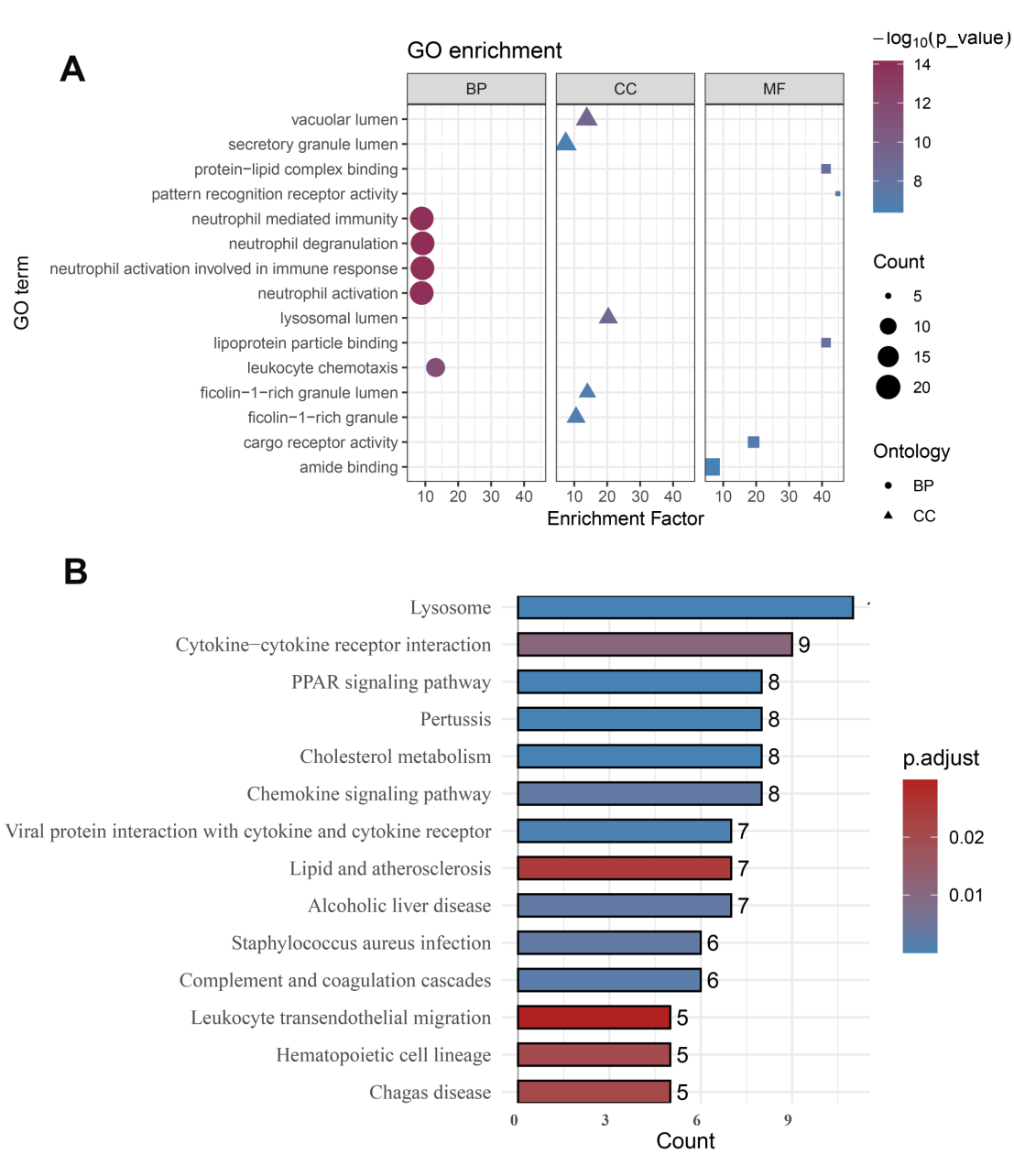


**Figure S4 Enrichment analyses of characteristics genes.** (A) GO analysis of the 91 genes. (G) KEGG analysis of the 91 genes.


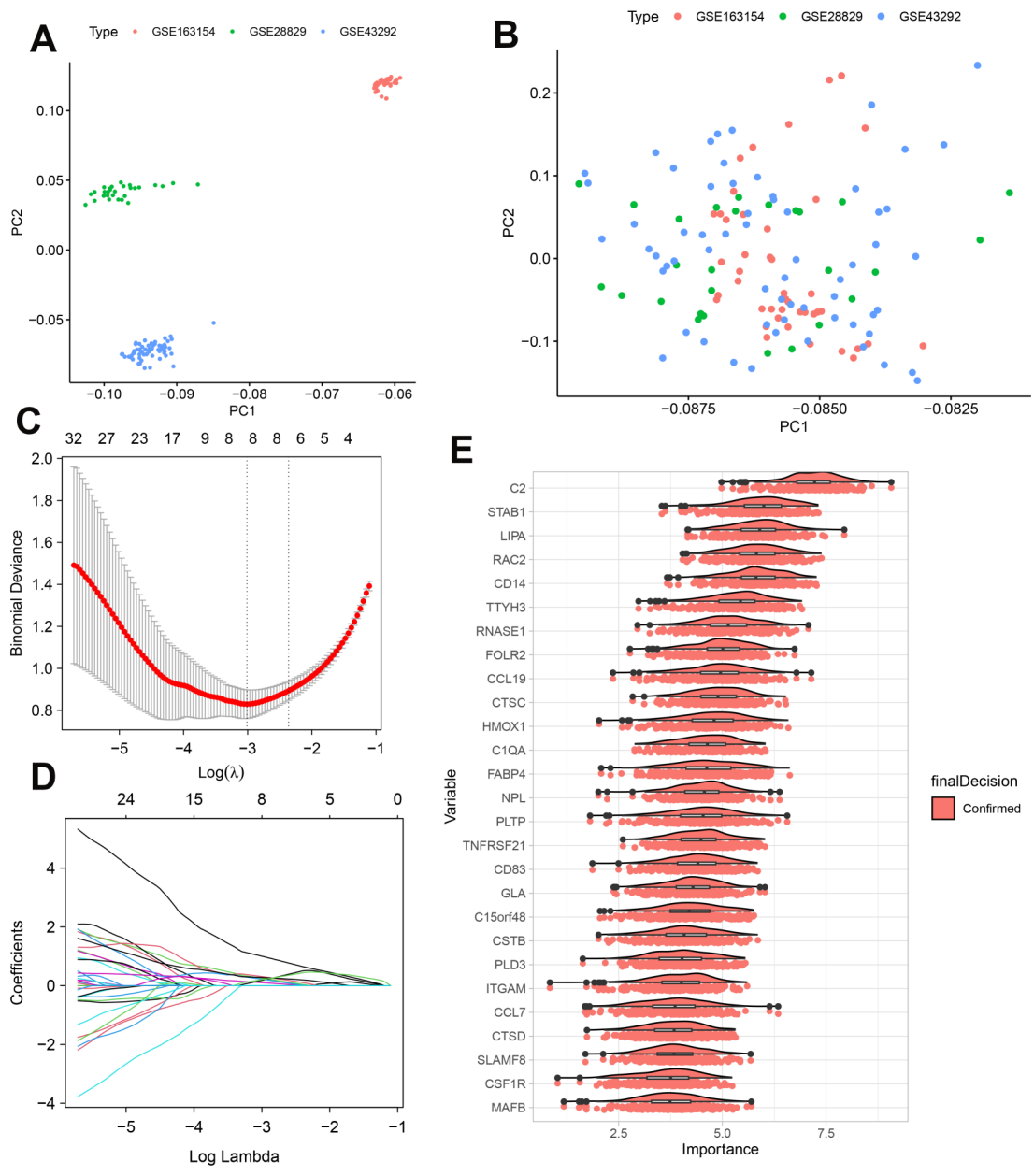


**Figure S5** (A)The distribution of the three datasets before combining.(B) The data distribution situation after the background calibration of the three data sets.(C)Tuning feature selection in the LASSO model. The vertical coordinate is the value of the coefficients, the subscript is log (lambda), and the superscript is the number of non-zero coefficients in the model at this point. (D) LASSO coefficient profiles of the feature genes. (E) The important variables identified by Boruta.


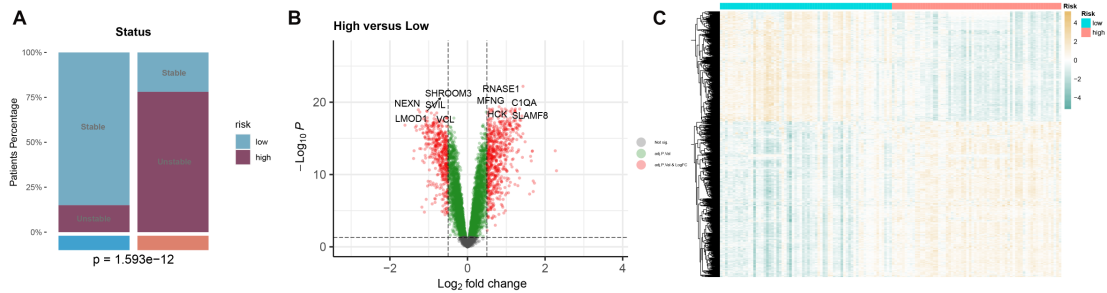


**Figure S6 Feature genes of high- and low-risk group**.


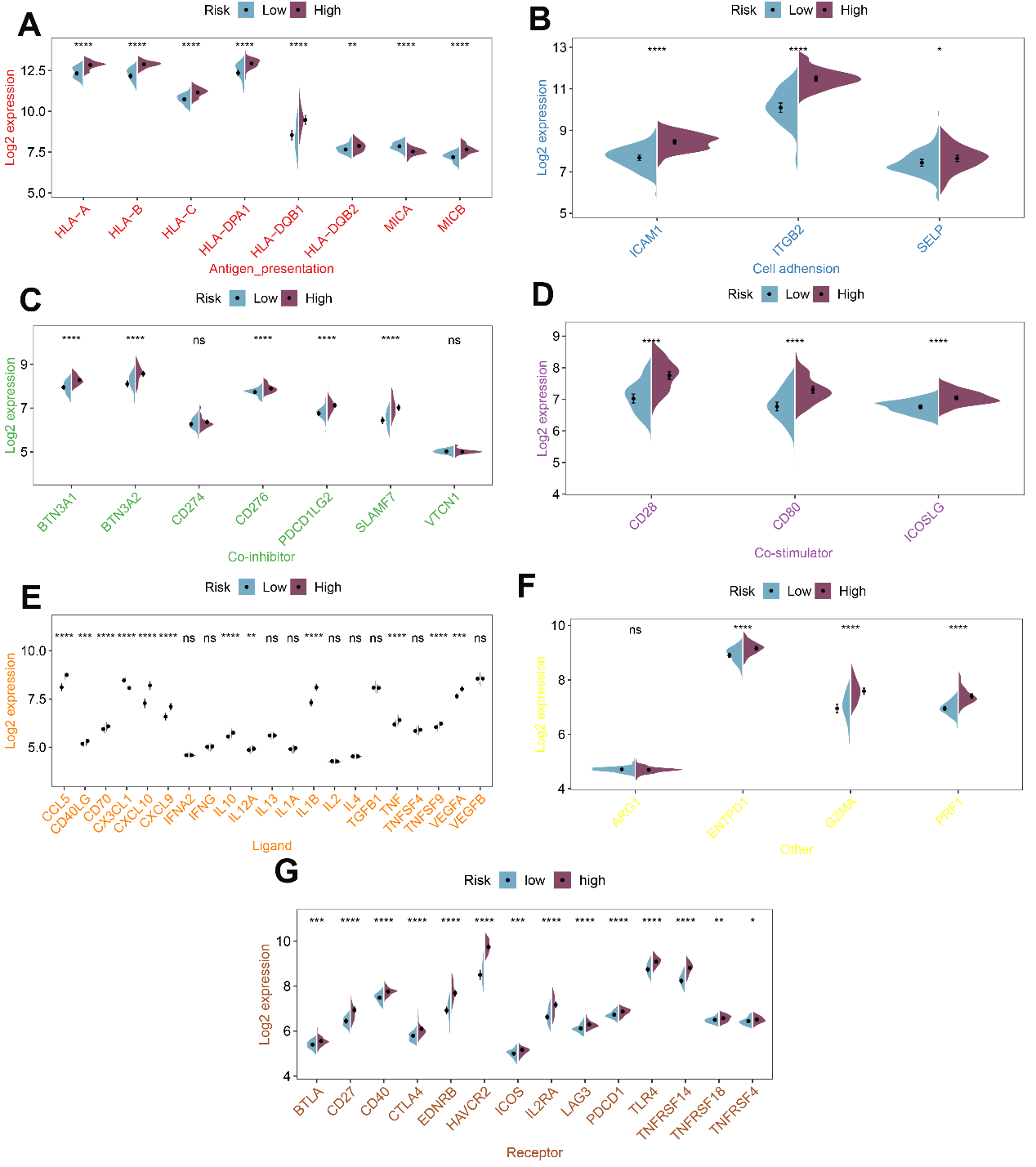


**Figure S7 the expression profiles of immunoregulatory subgroup genes in AS patients at low and high risk.** *p < 0.05, **p < 0.01, ***p < 0.001, ****p < 0.0001.
